# Age-dependent Pavlovian biases influence motor decision-making

**DOI:** 10.1371/journal.pcbi.1006304

**Published:** 2018-07-06

**Authors:** Xiuli Chen, Robb B. Rutledge, Harriet R. Brown, Raymond J. Dolan, Sven Bestmann, Joseph M. Galea

**Affiliations:** 1 School of Psychology, University of Birmingham, Birmingham, United Kingdom; 2 Max Planck University College London Centre for Computational Psychiatry and Ageing Research, London, United Kingdom; 3 Wellcome Centre for Human Neuroimaging, University College London, London, United Kingdom; 4 Sobell Department of Motor Neuroscience and Movement Disorders, Institute of Neurology, University College London, London, United Kingdom; Queen's University, CANADA

## Abstract

Motor decision-making is an essential component of everyday life which requires weighing potential rewards and punishments against the probability of successfully executing an action. To achieve this, humans rely on two key mechanisms; a flexible, instrumental, value-dependent process and a hardwired, Pavlovian, value-independent process. In economic decision-making, age-related decline in risk taking is explained by reduced Pavlovian biases that promote action toward reward. Although healthy ageing has also been associated with decreased risk-taking in motor decision-making, it is currently unknown whether this is a result of changes in Pavlovian biases, instrumental processes or a combination of both. Using a newly established approach-avoidance computational model together with a novel app-based motor decision-making task, we measured sensitivity to reward and punishment when participants (n = 26,532) made a ‘go/no-go’ motor gamble based on their perceived ability to execute a complex action. We show that motor decision-making can be better explained by a model with both instrumental and Pavlovian parameters, and reveal age-related changes across punishment- and reward-based instrumental and Pavlovian processes. However, the most striking effect of ageing was a decrease in Pavlovian attraction towards rewards, which was associated with a reduction in optimality of choice behaviour. In a subset of participants who also played an independent economic decision-making task (n = 17,220), we found similar decision-making tendencies for motor and economic domains across a majority of age groups. Pavlovian biases, therefore, play an important role in not only explaining motor decision-making behaviour but also the changes which occur through normal ageing. This provides a deeper understanding of the mechanisms which shape motor decision-making across the lifespan.

## Author summary

Decisions in everyday life often require weighing the probability of successfully executing an action (e.g., successfully crossing a street) against potential rewards and punishments. Although older individuals take fewer risks during such motor decision-making scenarios, the underlying mechanism remains unclear. Similar age-related changes in economic decision-making are explained by a decrease in Pavlovian attraction toward reward. However, despite the role of Pavlovian biases in linking action with reward and avoidance with punishment, their impact on motor decision-making is unclear. To address this, we developed a novel app-based motor decision-making task (n = 26,532). We found that motor decision-making was subject to Pavlovian influences. Although we found age-related changes for both punishment and reward-based decision-making processes, the most striking effect of ageing was a decrease in the facilitatory effect of Pavlovian attraction on action in pursuit of reward. Using data from an independent economic decision task in the same individuals (n = 17,220), we demonstrate similar decision-making tendencies for motor and economic domains across a majority of age groups. Hence, Pavlovian biases play an essential role in not only explaining motor decision-making behaviour but also the changes which occur through normal ageing.

## Introduction

Optimal decision-making requires choices that maximise reward and minimise punishment. Two key mechanisms play an important role in shaping the level of sub-optimality observed; a flexible, instrumental, value-dependent process, and a hard-wired, Pavlovian, value-independent process [1–3]. Choice behaviour in economic decision-making tasks has been widely studied and is often described using parametric decision models based on prospect theory that operationalise instrumental (value-dependent) concepts such as risk preference and loss aversion [4–7]. However, recent studies showed that changes in Pavlovian biases, which promote action towards reward and inaction in the face of punishment irrespective of option value [2, 8, 9], were able to account for aberrant choice behaviour during economic decision-making tasks. For example, the diminished economic risk-taking observed in older adults can be better explained by a reduction in dopamine-dependent Pavlovian attraction to potential reward [8, 10]. Importantly, value-dependent parameters based on prospect theory provide a poorer explanation of these changes in choice behaviour, suggesting that Pavlovian processes play a key role in the age-related changes observed during economic decision-making.

Motor decision-making, a unique type of decision-making, requires weighing potential rewards and punishments against the probability of successfully executing an action [11–14]. Motor decision-making has primarily been explained in the context of instrumental-based processes [14–19]. Within this framework, older adults display reduced risk-seeking behaviour [15]. However, given recent findings in economic decision-making [10], we asked whether Pavlovian biases might provide a more parsimonious explanation of age-related changes in motor decision-making. Although there is strong evidence that Pavlovian biases shape motor performance [20–23], and that healthy ageing leads to a reduction in Pavlovian biases on motor performance [24, 25], it is currently unknown whether Pavlovian biases influence motor decision-making. Sampling a large population through an app-based motor decision-making game, we provide a novel demonstration that Pavlovian biases have a substantial impact on motor decisions, and are able to account for age-related changes in choice behaviour during motor decision-making.

## Results

**Fig 1:**
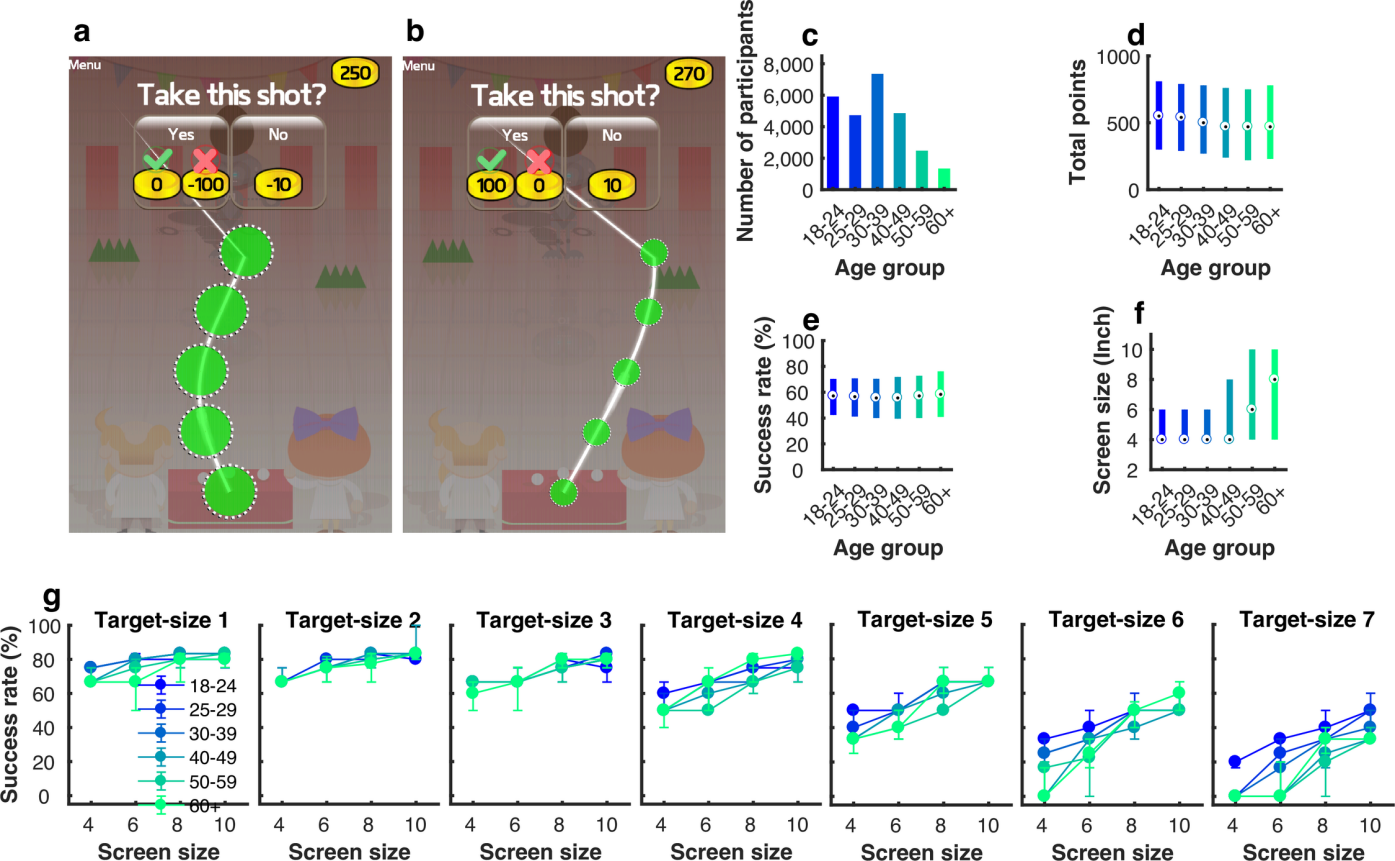
Motor gamble task and overall performance. (**a**) Game interface: an example of a punishment trial for target-size 1 (1: largest target size; 7: smallest target size); Participants decided whether to skip the tapping task and stick with a small punishment (-10 points) or gamble on successfully executing the action. If successful, they avoided the punishment (lose 0 points); otherwise, they received a greater punishment (-100 points); (**b**) A reward trial for target-size 7; (**c**) The number of participants in each age group; (**d**) Boxplots for the final points achieved; (**e**) The overall success rate for executing the tapping action; (**f**) The screen size (inches) of the devices used. The screen sizes were binned into [4 6 8 10 inches]. The central mark in the boxplots indicates the median, and the bottom and top edges of the thick lines indicate the 25^th^ and 75^th^ percentiles, respectively; (**g**) Success rate (%) for executing the tapping action given the age, the screen size, and target-size (1: largest target size; 7: smallest target size). Dots and error bars represent medians and bootstrapped 95%CIs.

We developed a novel app-based motor decision-making task that examined participant sensitivity to reward (gaining points) and punishment (losing points) when making a ‘go/no-go’ decision based on their perceived ability to successfully execute a motor action (Fig 1A and 1B). Using an app-based platform (‘How do you deal with pressure?’ The Great Brain Experiment: http://www.thegreatbrainexperiment.com/) [9, 26, 27], we obtained data from a large cohort (n = 26,532; 15,911 males; S1 Fig) in which six age groups were considered: 18-24yrs: n = 5889; 25-29yrs: n = 4705; 30-39yrs: n = 7333; 40-49yrs: n = 4834; 50-59yrs: n = 2452; and 60+yrs: n = 1319 (Fig 1C) [9, 26, 27].

The game required participants to sequentially tap 5 targets distributed along a pre-defined path that could vary in both curvature and direction (Fig 1A and 1B; see Methods). If a participant successfully tapped all 5 targets within 1.2 seconds, then the action was considered a success. There were 7 different target sizes, with the task becoming progressively more difficult as target size decreased (Fig 1A and 1B; see Methods). At the beginning of each trial, participants saw the required action (e.g., the target size and trajectory) and were asked whether they wanted to take the motor gamble. There were two types of trials: reward and punishment. For reward trials, participants had to decide whether to skip the trial and stick with a small reward (10 points) or gamble on successfully executing the tapping action (Fig 1B). If successful they received a greater reward (20, 60 or 100 points) or 0 points if they failed. For punishment trials, participants had to decide whether to skip the trial and stick with a small punishment (-10 points), or gamble on successfully executing the tapping action (Fig 1A). If successful, they avoided the small punishment (lose 0 points) but failure resulted in a greater punishment (-20, -60 or -100 points). Participants began with 250 points and the overall goal was to accumulate as many points as possible. All trial-by-trial data (including tasks parameters, behavioural results, modelling results and accompanying code) are available on our open-access data depository (https://osf.io/fu9be/).

We found that older adults won fewer total points than younger adults (Fig 1D; r = -0.056, 95%CI = [-0.069, -0.044], p<0.001; all age-related *r* values represent a partial correlation between the measurement of interest and age, whilst controlling for the effects of gender and education; the values in square brackets represent bootstrapped 95% CI; *p* values were computed by permutation tests; see Methods). The final points accumulated during this task were dependent on (1) the decisions made (to gamble or not) and (2) the motor performance (success rate of executing the tapping action). Therefore, prior to examining participant gambling choices it was crucial to determine whether motor performance differed across age groups.

Although success on the motor task was similar across age groups (Fig 1E; r = 0.009, 95%CI = [-0.003, 0.021], p = 0.142), older adults used devices with larger screen sizes than younger age groups (Fig 1F; r = 0.233, 95%CI = [0.221,0.245], p<0.001, S1 Fig). As target size was scaled to device screen size (see Methods), we assessed how the relationship between age, target size and screen size affected motor performance. We found that decreased success rate was linked to a combination of smaller target sizes, smaller screen sizes and older age (Fig 1G, multiple linear regression final model: success rate = 0.78–0.60 * targetsize + 0.10 * screensize −0.25 * age * targetsize + 0.24 * targetsize * screensize + 0.20 *age * targetsize * screensize; F(7,179043) = 6940, p<0.001, R^2^ = 0.213; all factors were normalized between 0 to 1). In addition, by examining the position of each tap relative to the centre of the 5 targets during successful trials, we found that all age groups showed a similar level of precision across levels and screen sizes (S2O–S2U Fig). However, there were clear differences in movement time (S2H–S2N Fig). Specifically, even during the easier levels, older adults moved significantly slower than younger adults (levels 1–3: r = 0.158, 95%CI = [0.154,0.162], p<0.001; partial correlation between movement time and age, whilst controlling for the effects of screen size). Therefore, this suggests that when the task became more difficult and required greater precision, older adults tended to fail more often (S2F and S2G Fig) because they were unable to reduce their movement time (given the time limit of 1.2 seconds). We next assessed choice behaviour in the context of how these factors influenced motor performance on a trial-by-trial basis.

**Fig 2:**
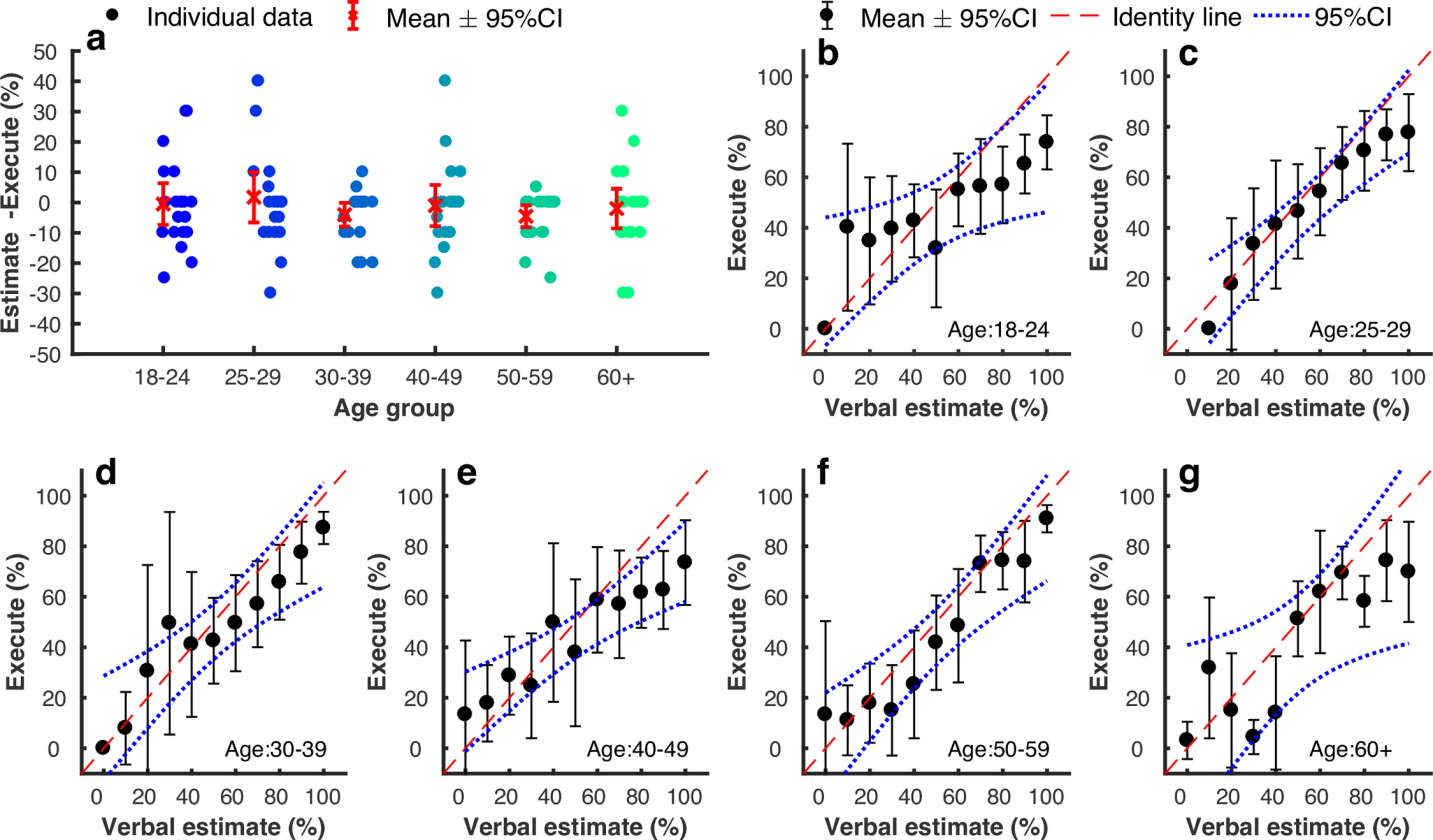
Participant’s ability to estimate motor performance success. **(a)** The average estimation error for each age group. Each participant’s estimation performance was evaluated as the average error across 42 trials (dots); the error on each trial was calculated as; estimate % - 100% if successful, 0% if failed. Red crosses and error bars represent the medians and 95%CIs across the participants within each age group; **(b-g)** For each age group (n = 120; 20 in each group), we calculated an average success rate for each available verbal estimate value (0% to 100% with 10% increment). Each black dot represents the median success rate (y-axis) across participants who gave that certain verbal estimate value (x-axis), and error bars represent bootstrapped 95% CI across participants.

As mentioned above, participants were asked to make decisions between a gamble option and a certain option. Each option can be characterised by its potential outcomes, weighted by the probability of each outcome (i.e. Expected Value [28]). For the gamble option, the expected value is given by: EV_gamble_ = P_success_V_success_+(1-P_success_)V_failed_, where P_success_ is the probability of successfully executing the tapping action; V_success_ is the points received if successful; V_failed_ is the points received on failure. The expected value of the certain option (EV_certain_) is V_certain_ as the probability of receiving this value is 1. We calculated P_success_ by estimating the probability of motor success based on a participant’s age, screen size of the device used and target-size level. Specifically, the probability of success for a participant within a certain age group, using a certain screen size and facing a certain target size on each trial was estimated using the average success rate across all the participants with the same age, same screen size, and facing the same target size (Fig 1G; see Methods). By comparing choice behaviour, given the difference in expected value between these two options (EV_gamble_-EV_certain_), we were then able to examine the influence of ageing on motor decisions while controlling for differences in motor performance due to age, screen size and target size. However, this formulation relied on an assumption that participants had a good estimate of their probability of success. To test whether this was true, we recruited an additional 120 participants (20 in each age group) who were asked to estimate their probability of success (from 0% to 100% in steps of 10%; see Methods) after being shown the target size and trajectory. After this estimate, they were then asked to perform the tapping action (whilst ignoring the decision-making part of the game). Each participant’s estimation performance was evaluated as the average error across 42 trials (Fig 2A). The error on each trial was calculated as: estimate % - 100% if successful, 0% if failed. Estimate performance did not differ across age groups (Fig 2A; one-way ANOVA: F(5,114) = 0.61, p = 0.695), and was not significantly different from zero (based on 6 t-tests, all p>0.05/6 = 0.0083). Therefore, similar to previous work [12, 14, 15], we found participants were able to estimate their probability of motor success reasonably well across all age groups (Fig 2B–2G). However, for all groups, the estimation performance was worst at extreme probabilities (Fig 2B–2G). This is in line with previous literature [29] and further explored with the use of weighted probabilities in the model (S1 Table).

**Fig 3:**
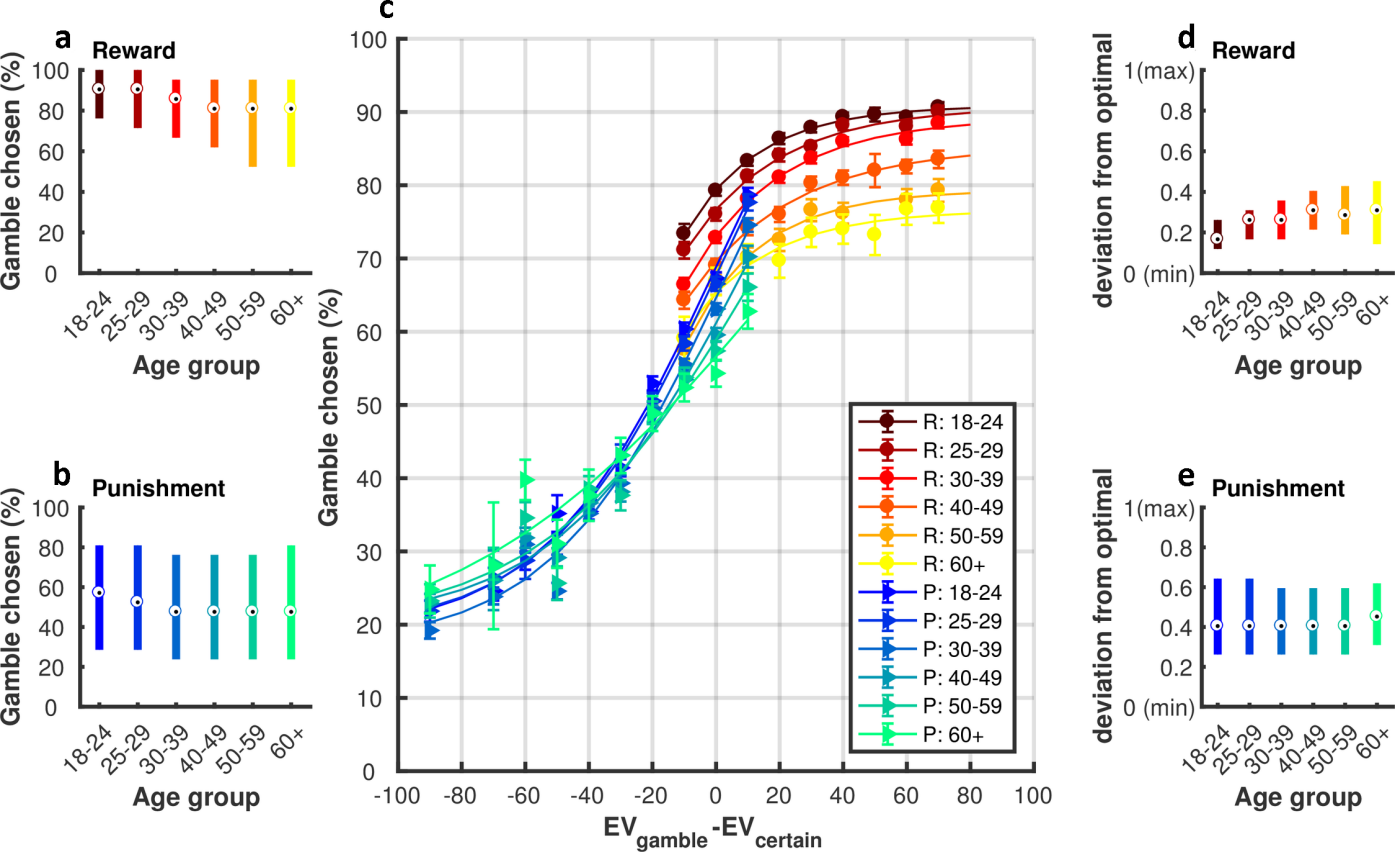
The proportion (%) of trials in which participants chose to gamble. (**a**) Gamble rate in the reward and **(b)** punishment domain. The central mark in the boxplots indicates the median, and the bottom and top edges of the thick lines indicate the 25^th^ and 75^th^ percentiles, respectively; (**c**) Propensity to choose the gamble option as a function of EVgamble−EV_certain_ (data was grouped into bin sizes of 10). As indicated in the legend, each of the warm colours represents one age group in the reward (R) condition, and each of the cool colours represents one age group in the punishment (P) condition. The lines are fitted lines to y = a*exp(-b*x)+c; R^2^ = 0.979 ± 0.022; Error bars represent bootstrap 95% CIs; **(d)** Discrepancy between choice behaviour and optimal decisions in the reward domain. Specifically, we calculated whether the optimal decision on each trial was to gamble (1 if EV_gamble_-EV_certain_>0) or skip (0, if EV_gamble_-EV_certain_<0). We then subtracted this value from the observed behaviour of the participant (gamble = 1, skip = 0). If the average absolute difference between these values across trials was 0, then a participant was deemed as an optimal decision-maker; **(e)** Discrepancy between choice behaviour and optimal decisions in the punishment domain.

We found a significant decrease in the proportion of trials in which participants chose to gamble across the lifespan in reward trials (Fig 3A; r = -0.186, 95%CI = [-0.198, -0.175], p<0.001), and to a lesser extent in punishment trials (Fig 3B; r = -0.053, 95%CI = [-0.065, 0.041], p<0.001). To understand these results, we examined age-related changes in choice behaviour given the difference between the options (EV_gamble_-EV_certain_). Interestingly, in reward trials (warm-coloured-dotted lines in Fig 3C), there was a gradual and monotonic decrease in gamble rate across the lifespan which appeared to be independent of the EV_gamble_-EV_certain_ value. In contrast, for punishment trials (cool-coloured-triangle lines in Fig 3C), older adults displayed a higher gamble rate during high risk gambles (e.g., EV_gamble_-EV_certain_ = -90, where green lines (older adults) are above the blue lines (younger adults)), but conversely a relatively lower gamble rate during low risk gambles (e.g., EV_gamble_-EV_certain_ = 0 where green lines are below the blue lines).

Given these results, do older adults make worse motor decisions? An ideal (optimal) decision-maker chooses the option that has the higher expected value, and we therefore compared participant choice behaviour with the optimal behaviour. Specifically, we calculated whether the best decision on each trial was to gamble (if EV_gamble_-EV_certain_>0, coded as 1) or decline (if EV_gamble_-EV_certain_<0, coded as 0). We then subtracted this value from the observed choice of the participant (also coded gamble = 1, decline = 0). If the average absolute difference between these values across trials was 0, then a participant was deemed an optimal decision-maker. In reward trials, there was progressive deviation from optimality across the lifespan (Fig 3D; r = 0.258, 95%CI = [0.245,0.270], p<0.001). In contrast, for punishment trials, all age groups showed a similar level of sub-optimality (Fig 3E; r = 0.001; 95%CI = [-0.011, 0.013], p = 0.832). Therefore, the most pronounced effect of ageing on motor decision-making was a value-independent decrease in gamble rate during reward trials which led to a significant decrease in optimality.

**Fig 4:**
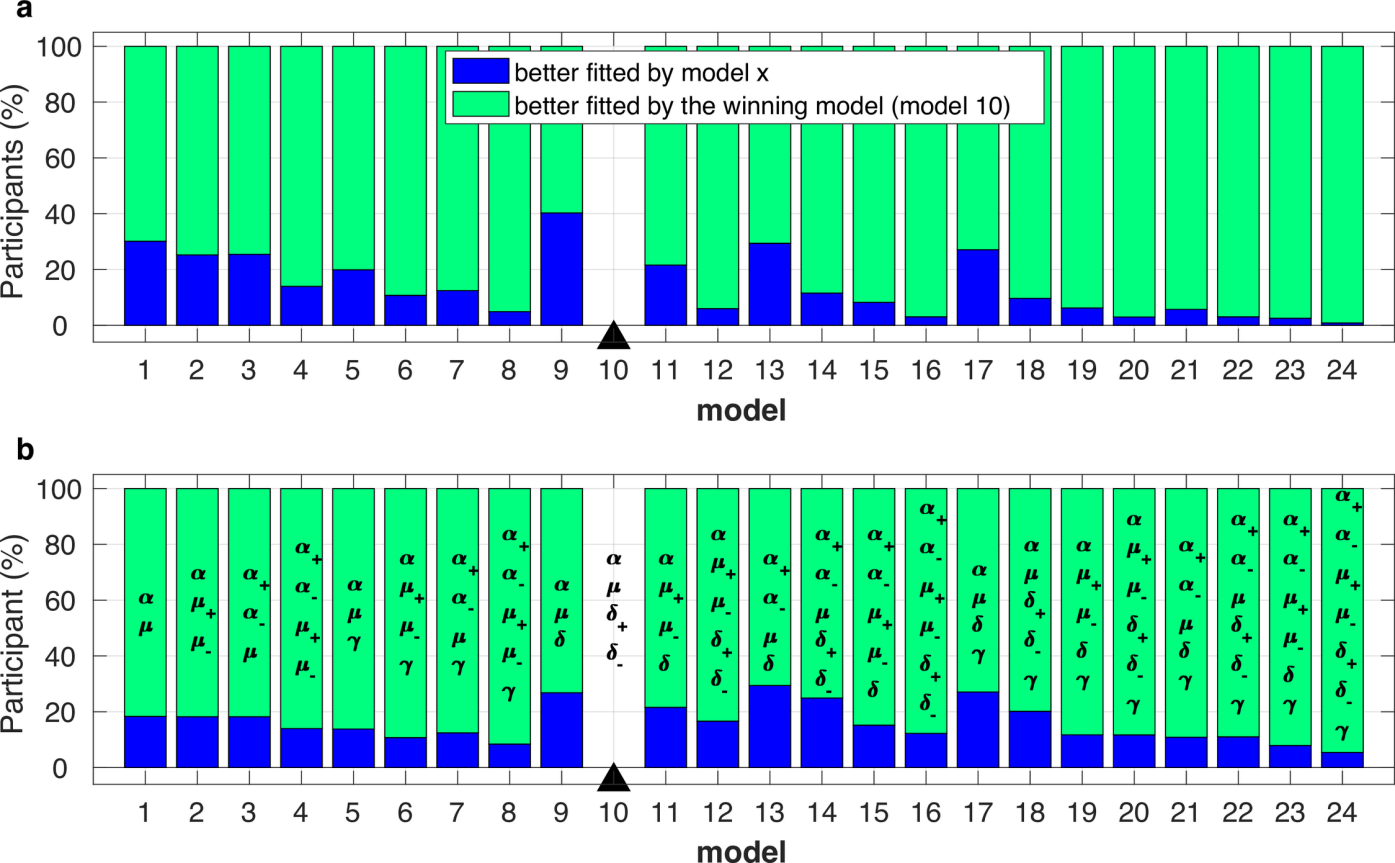
Subject-level AIC and BIC model comparison. **(a)** For each participant, the winning approach avoidance model’s (model 10 [α, μ, δ+, δ−] as indicated by the black triangle; this model had the smallest summed BIC; S1 Table) BIC value was compared with the BIC value for each of the other models (x-axis). The percentage (%) of participants for which the winning model (smaller BIC value is preferred) better fitted their choice behaviour is shown in green (upper part of the bar). The percentage of participants for which the alternative model better fitted their choice behaviour is shown in blue (lower part of the bar). **(b)** Identical analysis for AIC. Parameter set for each model is also provided.

**Fig 5:**
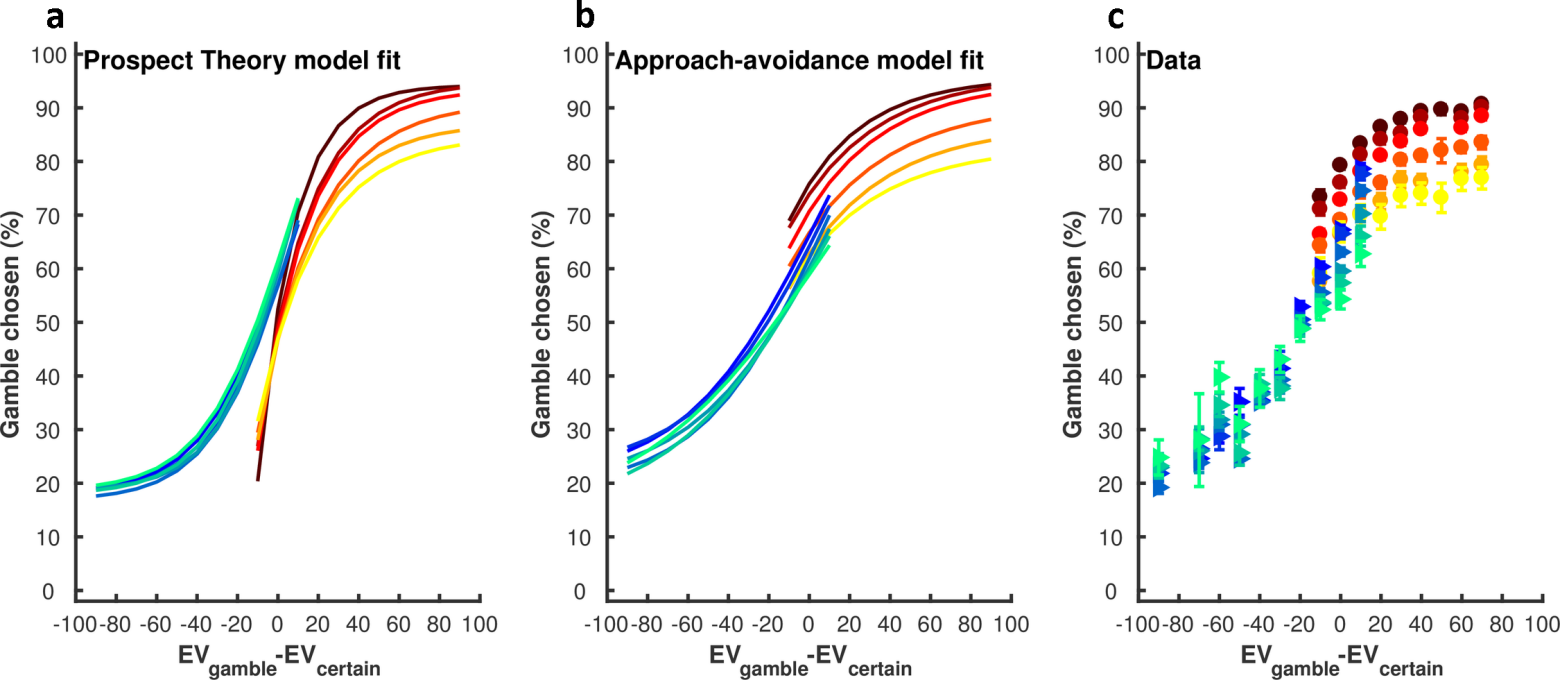
Average model fits across participants. **(a)** Average model fit for the winning prospect theory model (ID = 4 in S1 Table [α**^+^**,α**^−^**,μ**^+^**,μ**^−^**]). The probability of choosing the gamble option (y-axis) predicted by the model (see Methods; Eq 2) is plotted against the difference in expected value between the two options (x-axis; EV_gamble_−EV_certain_; grouped into bin sizes of 10). As indicated in the legend, each of the warm colours represents one age group in the reward (R) condition, and each of the cool colours represents one age group in the punishment (P) condition. The model cannot account for the observed differences across the life span, including (1) the gradual and monotonic decrease in gamble rate across the lifespan in the reward domain; (2) the changes in gamble propensity observed in the punishment domain across age groups (as the model fit shows almost no difference across the age groups in the punishment domain); (3) the higher gamble rate in the reward domain relative to the punishment domain when [EV_gamble_-EV_certain_] is close to 0 (as the model fit shows the opposite) (model falsification [33]); **(b)** Average model fit across participants for the winning approach-avoidance model (ID = 10 in S1 Table, [α,μ, δ**^+^**,δ**^−^**]); **(c)** Data: propensity to choose the gamble option as a function of EV_gamble_−EV_certain_.

While these data portray many similarities with decision-making under risk [4, 5], there are also clear differences. For example, decision-making models based on prospect theory are not able to explain the gradual, monotonic and value independent decrease in gamble rate across the life span observed during the reward trials [8] (Fig 3C). We predicted that such dichotomies represented the contribution of value-independent Pavlovian approach-avoidance biases to motor decision-making behaviour. To test this prediction, we modelled the choice behaviour using an established decision-making model based on prospect theory, and a newly introduced model that included Pavlovian approach-avoidance parameters [8, 10] (see Methods). For the prospect theory parametric decision-making model, the utility of the gamble option is given by U_gamble_ = P_success_ x (V_gamble_)^alpha^; and the utility of the certain option is U_certain_ = (V_certain_)^alpha^, where the risk preference parameter (alpha: α) represents the diminishing sensitivity to change in value with an increase in absolute value (S3 Fig). The gamble probability is then a stochastic function of value that is described by the softmax function: P_gamble_ = (1+exp(-μ(U_gamble_- U_certain_)))^-1^. The logit parameter μ is the sensitivity of the choice probability to the value difference. In addition to these parameters (α,μ), the Pavlovian approach-avoidance model included a value-independent parameter (δ). Specifically, gamble probability is further decided by the parameter δ: P_gamble_ = (1+exp(-μ(U_gamble_- U_certain_)))^-1^+δ. Positive or negative values of the Pavlovian parameter correspond, respectively, to an increased or decreased probability of gambling without regard to option values (see Methods; Eq 3). According to Akaike’s information criterion (AIC) [30], Bayesian information criterion (BIC) [31] model comparison (Fig 4; S1 Table; see Methods) and model/parameter recovery analysis (S4 Fig; see Methods), we found that an approach-avoidance decision model with 4 parameters (a joint risk preference parameter: *α*, the inverse temperature parameter: *μ*, value-independent parameters exclusively for reward *δ*^+^ and punishment *δ*^−^ trials) fitted the motor gamble (choice) data better than other decision models (Fig 5; see Methods). To confirm this result, we performed a repeated-measures ANOVA across all participants using the log-evidence (AIC/BIC) as a summary statistic for each model [32]. The ANOVA showed a significant difference between models (BIC: F_(23,529)_ = 5085.9, p<0.001; AIC: F_(23,529)_ = 4011.6, p<0.001). Posthoc paired t-tests (with Bonferroni correction for multiple comparisons) showed that AIC/BIC values for the winning model ([α, μ, δ+, δ-]; S1 Table) were significantly lower (lower value = preferred model) than for all other models (p<0.001; Fig 4).

**Fig 6:**
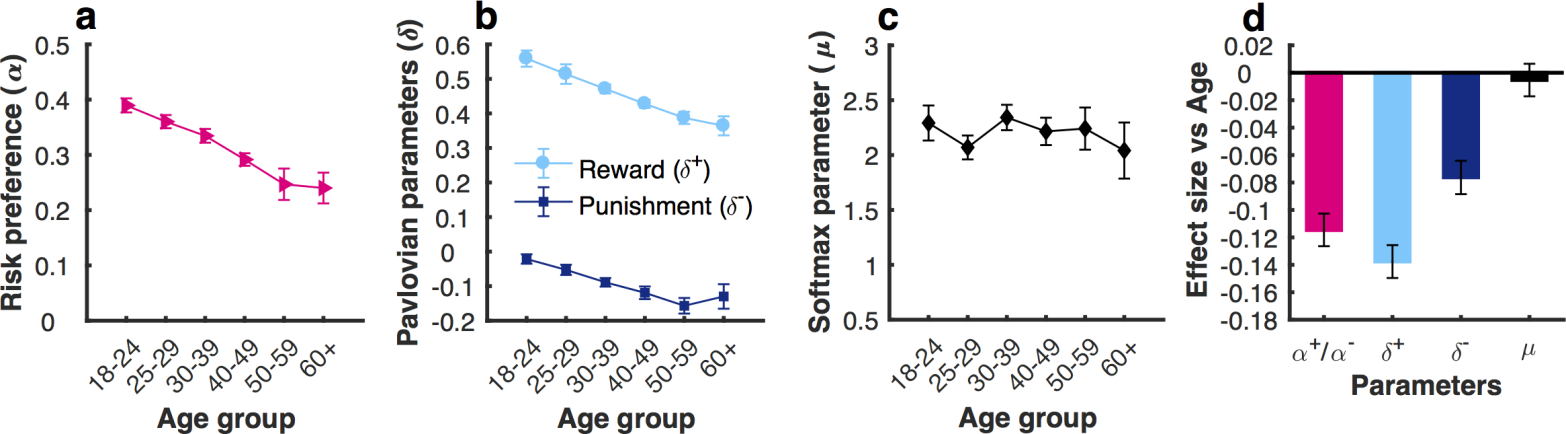
The change in approach-avoidance model parameters across the life span. (**a**) α across age groups; (**b**) δ**^+^** and δ**^−^** across age groups; (**c**) μ across age groups; (**d**) Age-related decline for the parameters. The largest effect size was observed for the Pavlovian approach parameter (δ**^+^**). This age-related effect was not observed for the temperature parameter, μ. Error bars represent 95%CI.

Based on the preferred approach-avoidance decision model ([α, μ, δ+,δ-]; S1 Table), we observed age-related changes across the reward and punishment domains for both risk preference and Pavlovian parameters. However, the most striking effect was a decrease in the facilitatory effect of Pavlovian attraction on action in pursuit of reward (*δ*^+^). Specifically, we found that healthy ageing did not affect the stochasticity parameter, μ (r = -0.005, 95%CI = [-0.017, 0.0065], p = 0.390), but was associated with a decrease in the risk preference parameter, *α* (Fig 6A and 6D; r = -0.115, 95%CI = [-0.126,-0.103], p<0.001). The winning model included a α parameter, but this represented different value-dependent biases in reward and punishment (*α*<1 indicated risk aversion in reward domain and *α*<1 represented risk-seeking in punishment domain; *α* = 1 represented risk-neutral; see Methods). Therefore, the reduction in the alpha parameter value across age groups reflected reduced sensitivity to the value change, resulting in greater risk-aversion in reward and risk-seeking in punishment. However, the enhanced risk-seeking in the punishment domain was offset by the fact that ageing was also linked with greater Pavlovian avoidance, causing a reduction in gamble rate (Fig 6B and 6D; *δ*^−^; r = -0.076, 95%CI = [-0.089,-0.064], p<0.001), an effect not previously observed in economic decision-making [10]. Nevertheless, the largest impact of ageing was a substantial decrease in Pavlovian attraction (Fig 6B and 6D; *δ*^+^; r = -0.138, 95%CI = [-0.150,-0.126], p<0.001). We found a similar decline for both sexes (S5 Fig; male: n = 15911, r = -0.134, 95%CI = [-0.154,-0.116], p<0.001; female: n = 10621, r = -0.140, 95%CI = [-0.156,-0.124], p<0.001), and across all education levels (S6 Fig; school: n = 9171, r = -0.146, 95%CI = [-0.171,-0.122], p<0.001; university: n = 11281, r = -0.129, 95%CI = [-0.148,-0.111], p<0.001; advanced: n = 6080, r = -0.125, 95%CI = [-0.151,-0.100], p<0.001). Importantly, we did not observe this age-related effect for the temperature parameter (μ; Fig 6D), indicating the changes observed in the risk and Pavlovian parameters were not simply a result of large participant numbers.

**Fig 7:**
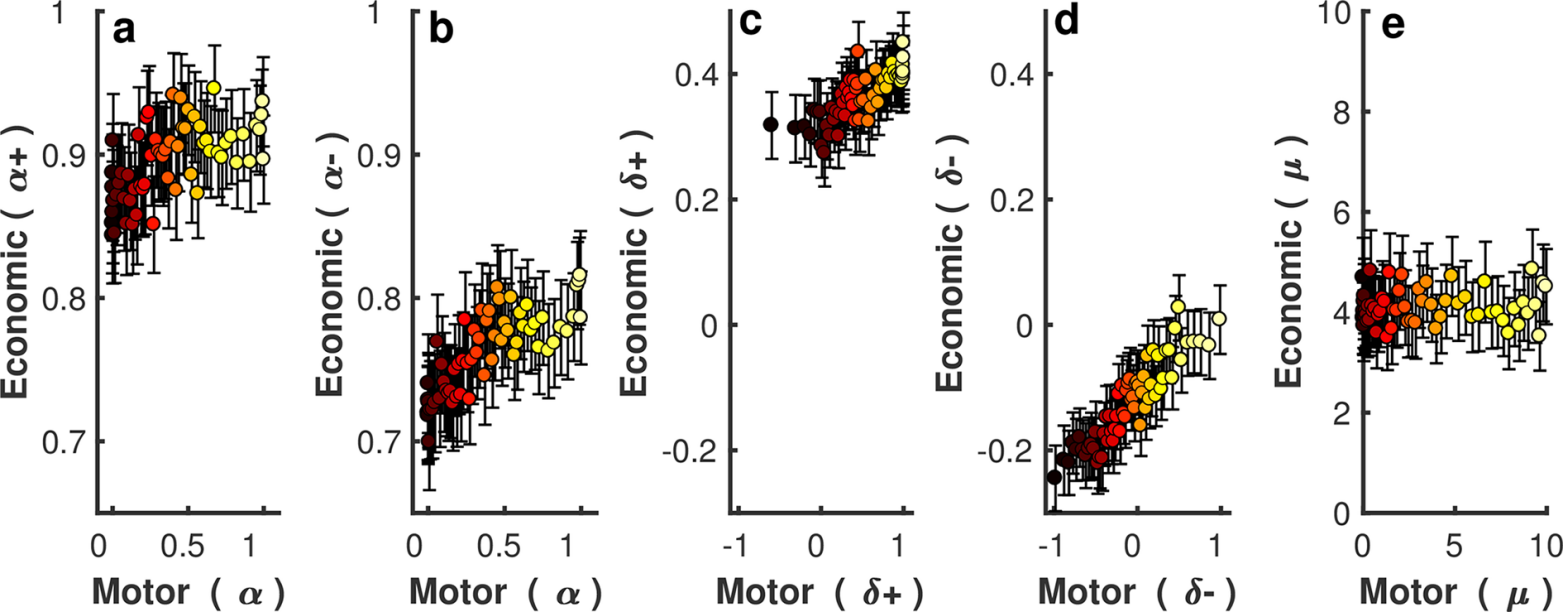
The relationship between approach-avoidance model parameters across the motor (x-axis) and economic (y-axis) gambling tasks. **(a-e)** Each panel represents a single parameter; (a)The risk preference parameter in reward; and **(b)** punishment trials; **(c)** Pavlovian approach-avoidance parameter in reward; and **(d)** punishment trials; **(e)** the temperature parameter. The participants were binned into 60 groups (287 participants in each group) based on their motor parameter value. Within each panel, each dot represents the medians of motor parameters (x-axis) and economic parameter (y-axis) for each group. Error bars represent 95%CI in the economic decision-making task.

Finally, through this app-based platform a subset of participants (n = 17,220) also performed an economic decision-making gambling task in which a similar approach-avoidance model was used to explain choice behaviour [10] (S7 Fig). The data and model have previously been published [10]. Briefly, this work compared a single approach-avoidance ([α+, α-, μ, δ+, δ-]) and prospect theory model ([α+, α-, μ]), with the approach-avoidance model fitting the data better [10]. In addition, analysis of laboratory data using a similar economic decision-making task confirmed that the approach-avoidance model outperformed reasonable alternative models including simpler models based on prospect theory [8]. As the economic decision-making app data revealed a similar age-related decline in the Pavlovian approach parameter [10] (S7 Fig), we compared the subset of participant’s, who performed both tasks, parameter values across the two winning approach-avoidance models. A small yet significant positive relationship was found between the tasks for risk preference (Fig 7A and 7B; r(α+) = 0.05, 95%CI = [0.04,0.07], p<0.001; r(α-) = 0.08, 95%CI = [0.06,0.09], p<0.001) and Pavlovian parameters (Fig 7C and 7D; r(δ^+^) = 0.07, 96%CI = [0.06,0.09], p<0.001; r(δ^−^) = 0.14, 95%CI = [0.12,0.15], p<0.001). However, we did not observe this correlation for the temperature parameter (Fig 7E; r(μ) = 0.004, 95%CI = [-0.01,0.02], p = 0.587). This relationship was relatively consistent within the first 5 age groups (S8 Fig, S9 Fig). However, although the oldest age groups (60+) showed a similar trend, the positive correlation was not significant (S8 Fig, S9 Fig). Within this age group, it is possible that we did not have enough power (participant numbers) to reliably detect a significant correlation given our observed effect sizes. Specifically, the 60+ age group (n = 663) achieved 0.3–0.69 power (desired level is usually >0.80) to detect a significant correlation across the 4 parameters (S2 Table).

## Discussion

Making decisions under uncertainty is crucial in everyday life, whether it is managing retirement funds, choosing a career, or deciding between pulling out or not on to a busy road whilst driving. The latter example describes motor decision-making, a unique kind of decision that requires weighing potential rewards and punishments against the probability of successfully executing an action [11–14], and often with immediate outcomes. Although healthy ageing has been associated with reduced risk taking during motor decision-making [15], the underlying mechanism was not clear. We contributed answers to this question using a novel motor gambling task that exploited an app-based platform. This enabled us to collect a large cohort of data. Unlike previous work on motor decision-making [11–14], we considered choice behaviour in relation to both value-dependent instrumental and value-independent Pavlovian processes [2, 8, 9]. We found age-related changes across the reward and punishment domain for both value-dependent and independent parameters. However, the most striking effect of ageing was a decrease in the facilitatory effect of Pavlovian attraction on action in pursuit of reward. Through this app-based platform, we also compared a subset of participant choice behaviour on the motor decision-making task and a separate economic decision-making task [10]. We found similar decision-making tendencies across motor and economic domains.

Our large cohort and use of a newly established approach-avoidance computational model [10, 34] enabled us to detect subtle age-related changes in choice behaviour and surprising interactions between value-independent and value-dependent processes. For instance, the risk aversion parameter (*α*: instrumental value-dependent process) was on average less than 1 across all age groups, indicating risk aversion in reward, and risk seeking in punishment. The use of a joint risk preference parameter (alpha) across reward and punishment signified a similar degree of value-dependent bias across gain and loss trials (risk aversion in reward and risk-seeking in punishment). This is in contrast to the economic decision-making data (10) in which separate alpha values were used, signifying subtle differences across the tasks. Importantly, this parameter progressively decreased with age, suggesting that older adults showed increasing value-dependent biases, i.e., more risk aversion in reward and more risk seeking in punishment. This is in line with previous economic decision-making work which revealed older adults weigh certainty (achieving the small reward or avoiding the small punishment) more heavily than younger adults [35]. Interestingly, the greater risk-seeking in the punishment domain was offset by the fact that ageing also led to greater Pavlovian avoidance, an effect not observed in economic decision-making [10]. It is the interaction between value-dependent and independent parameters that help explain not only the complex changes observed with ageing during punishment trials but also the lack of difference across age groups for punishment-based optimality. Crucially, previous work in motor decision-making using only instrumental-based processes would not have detected such complex behavioural interactions [13–15]. The underlying mechanism behind age-dependent increases in Pavlovian avoidance is unknown. It has been suggested that the neurobiology behind Pavlovian avoidance may involve opponency between the dopaminergic and serotonergic systems [36]. Despite there being evidence of age-related decline in serotonin receptor availability [37], it remains an open question as to the link between serotonin and Pavlovian avoidance during either motor or economic decision-making.

The strongest effect of ageing was a decrease in Pavlovian attraction to reward. As all age groups displayed risk aversion during reward trials, this decrease in Pavlovian attraction led to reduced optimality in older adults. These results are strikingly similar to the ones observed in economic decision-making [10], suggesting Pavlovian attraction plays a pivotal role in explaining age-related changes to reward across both motor and economic decision-making. During economic decision-making, boosting dopamine with L-DOPA increases the influence of Pavlovian attraction on choice behaviour [8]. In addition, healthy ageing is associated with a gradual decline in dopamine availability [38, 39] and neural responses to reward [40]. Therefore, it is possible that the decrease in Pavlovian attraction during motor decision-making in older adults is a result of an age-dependent decrease in dopamine availability.

More broadly, the current work shows the importance of both instrumental value-dependent and Pavlovian value-independent processes in motor decision-making. However, task design may play an important role in determining the size of Pavlovian influences. Here we used a ‘go/no-go’ decision-making task as previous literature has shown the ‘go/no-go’ component induces strong Pavlovian biases [2, 22, 41]. It remains to be seen whether computational models including Pavlovian biases provide a better description of choice behaviour during other motor decision-making tasks which do not involve a ‘go/no-go’ component [14–19].

We found that older participants gambled less frequently in both gain and loss trials. Although we modelled this change with Pavlovian approach-avoidance, this decrease in overall gamble rate could also be explained by other value-independent factors that make the gamble option less ‘attractive’ for the older population such as an increased sensitivity to the cost of physical effort [42], age-related changes in motor performance or an age-related underestimation of movement success. However, one critical feature of Pavlovian approach-avoidance is that it has opposite effects on gain and loss trials, i.e., ‘approach’ in gain trials and ‘avoidance’ in loss trials. This is the main reason that the approach-avoidance model was able to explain data significantly better than the prospect theory model. In particular, across age groups, participants gambled more often in the gain trials relative to loss trials when the EV_gamble_—EV_certain_ was close to zero. Such a result cannot be explained without an opposing effect on gain and loss trials, which shifts gamble rate higher for gain trials and lower for loss trials. To our knowledge, there is no evidence to suggest that an age-related change in effort cost, motor performance or estimation of movement success would have such opposing effects on gain and loss trials.

Finally, participants showed similar decision-making tendencies for both instrumental (value-dependent) and Pavlovian (value-independent) parameters across motor and economic domains for all age groups except 60+. At present, we are unsure whether the non-significant correlation in the 60+ age group is a true reflection of motor and cognitive decisions becoming dissociated with age, or simply a lack of power within this age group to observe small effect sizes. Despite this, the general association between cognitive and motor decision-making extends previous work that revealed a similar relationship with parameters derived from parametric decision models based on prospect theory [14, 16] and reinforces the view that the mechanisms which control cognitive (economic) and motor decision-making are somewhat integrated [43]. However, the correlation between the tasks was small, around r = 0.1, suggesting that while participants showed similar behavioural trends across the two tasks, their performance in motor and economic domains was also distinct [16]. Interestingly, the approach-avoidance model not only fitted choice data substantially better for the motor decision-making task, relative to the economic task, but the effect size relating to age was also nearly double in size for all parameters [10]. This indicates that while there are clear similarities between cognitive and motor decision-making, computational models including Pavlovian biases appear to be particularly important for explaining motor decision-making. Despite their role in linking action and reward and inaction with punishment, descriptions of Pavlovian biases are surprisingly absent in current computational models of motor function.

In conclusion, Pavlovian biases play an important role in not only explaining motor decision-making behaviour but also the changes which occur through normal ageing. This provides a greater understanding of the processes which shape motor decision-making across the lifespan, and may afford essential information for developing population wide translational interventions such as promoting activity in older adults.

## Methods

### Participants

We tested 26,532 participants (15,911 males, aged 18–70+) who completed the task between November 20, 2013 and August 15, 2015. Data were only included if users fully completed the game and it was their first attempt. All participants gave informed consent and the Research Ethics Committee of University College London approved the study. We also recruited an additional 120 participants (49 males, aged 18–70+) who were asked to estimate their success rate (motor performance). These participants were mainly recruited by email advertisement sent to staff and students at the University of Birmingham.

### Materials and apparatus

Using an app-based platform (The Great Brain Experiment: www.thegreatbrainexperiment.com) we developed a motor decision-making task (‘How do I deal with pressure?’) which is freely available for Apple iOS and Google Android systems. The game runs in a 640x960 (3:4 ratio) pixel area, which is then scaled to fit the screen whist maintaining this ratio. The game required participants to ‘throw’ a ball at a coconut in an attempt to knock it off its perch. This was achieved by tapping 5 sequential targets along a pre-defined path. The path was characterised by an angle parameter that represented a section of a sine curve, in degrees. The curves were drawn from the bottom (the starting point) to top of the game window (Fig 1A and 1B). For example, if the angle parameter was 360, then one complete cycle of the sine curve was used to draw the curve. During the task, the angle was randomly chosen between 0 and 360. The 5 targets were evenly spaced along the curves. If the participant tapped all 5 targets sequentially (from bottom to top) within 1.2 seconds, then the action was considered a success and the coconut was hit. If the participant failed to tap all 5 targets accurately or within the allotted time then the action was considered a failure and the ball sailed past the coconut. In addition, the action was deemed a failure if participants did not start the tapping action within 7 seconds after they chosen to do the tapping. There were 7 different target sizes across trials with the tapping action becoming more difficult as the target size was reduced. However, as mentioned above, the game interface was scaled to screen size. Therefore, motor performance (success rate) was examined relative to the interaction between target size and screen size (Fig 1G). All trial-by-trial data (including tasks parameters, behavioural results, modelling results and accompanying code) are available on our open-access data depository (https://osf.io/fu9be/).

### Motor gambling task

At the beginning of each trial, participants were shown the action required (i.e. the position and size of the 5 targets) and were asked to make a motor gamble. There were two types of trials: reward and punishment (Fig 1A and 1B). For reward trials, participants had to decide whether to skip the trial and stick with a small reward (10 points) or gamble on successfully executing the ‘throw’. If successful they received a greater reward (20, 60 or 100 points) but 0 points if they failed. For punishment trials, participants had to decide whether to skip the trial and stick with a small punishment (-10 points) or gamble on successfully executing the ‘throw’. If successful they lost nothing (0 points) but failure resulted in a greater punishment (-20, -60 or -100 points). Hence, there were 6 value combinations. Each combination was repeated for each of the 7 different target sizes (6 values x 7 target sizes = 42 trials). Although there were 7 blocks of the game this did not directly relate to the 7 target sizes. In order to maintain a level of unpredictability, the first 3 blocks included random presentation of the 3 largest (easiest) target sizes, the next 3 blocks included target sizes 4–6 and the final block included the smallest (most difficult) target size. Participants began with 250 points and the overall goal was to accumulate as many points as possible.

For the control study (Fig 2), which examined participant ability to estimate their probability of success, individuals were asked to estimate their probability of motor success (0% to 100% in steps of 10%) after being shown the target size and trajectory. After this estimate, they were then asked to perform the tapping action, whilst ignoring the decision-making part of the game. This experiment was not performed in the laboratory but across campus at the University of Birmingham with the first 60 participants (10 per group) using their own mobile device and the second 60 participants (10 per group) using a device provided with a screen size of 5.1 inches. Screen sizes of the devices used for first set of 60 participants had a similar profile as in the main experiment (Fig 1 and S1 Fig). Importantly, we did not find a significant difference in estimate performance with (M = -0.023, SD = 0.126) or without (M = -0.011, SD = 0.139) screen size control (t(118) = -0.515, p = 0.607; S10 Fig).

### Data analysis

Matlab (Mathworks, USA, 2016a) was used for all data analysis. We report partial correlation coefficients (r) for the relationships between the measures of interest (e.g., final points achieved, screen size of the devices, sub-optimality, model parameters) and age, whilst controlling for the effects of gender and education. Specifically, the partial correlation between *X* (e.g., screen size) and *Age* given the controlling variables *Z* = [gender, education] was given by the correlation between the residuals *eX* and *eAge* resulting from the linear regression of *X* with *Z* and of *Age* with *Z*, respectively. These were implemented using the Matlab function ‘partialcorr’. Spearman rank correlation was used as the participants were asked to identify themselves into one of the age groups provided, therefore the variable *Age* was ordinal. Bootstrapped 95% confidence intervals (CI) were computed based on 10,000 resamples with replacement with the bias-corrected and accelerated (BCA) bootstrap method. This was implemented using the matlab function ‘bootci’. All p values were computed based on permutation tests using 10,000 random shuffles of age labels to determine null distributions [10]. Bootstrapped and resampling techniques were used because (1) previous studies have shown that the coverage of bootstrapped Cis is as good or better than coverage of analytic CIs, especially when using Spearman’s correlation with ordinal data [44] and (2) Bootstrapping and resampling techniques, based on recent work [45], have proven to be a robust alternative to analytic statistical techniques.

### Parametric decision-making models

On each trial participants faced a gamble that contained a certain option involving a payoff of certain points (+10 in reward trials and -10 in punishment trials), and a gambling option in which the outcome depended on a probability of successfully executing the tapping action. This probability was estimated given a participant’s age, screen size of the device used and target-size level (Fig 1G). Specifically, the probability of success for a participant within a certain age group, using a certain screen size and facing a certain target size on each trial was estimated using the average success rate across all the participants with the same age, same screen size, and facing the same target size. Given the small amount of trials we had for each participant at each target size to estimate their probability of success, we believed this group average approach was the most valid estimate of success probability. However, we also conducted the analysis when success probability was estimated based on each individual’s own data (i.e. the probability of success for a participant facing a certain target size was estimated using their own success rate over the same target size). Importantly, our findings still hold (S11 Fig). We modelled participant motor gamble choices using an established decision-making model based on prospect theory [5] and a newly introduced model which included an extra Pavlovian approach-avoidance [8, 10, 34] component. In the following, we first describe the prospect theory models, followed by the approach-avoidance models.

Parametric decision-making model based on prospect theory: There are three key components in prospect theory models. The first component is the value function. According to prospect theory, the subjective desirability of outcomes is modelled as transformations of objective task quantities. The subjective desirability of the outcomes, *v*(*O*) was modelled by a value function (2-part power function) of the form:

$$v(O)=\left\{ \begin{array}{ll} O^{\alpha } & if\;O\geq 0\\ -\lambda ∙({-O)}^{\alpha } & if\;O<0 \end{array}\right.$$ Eq 1

where, the risk preference parameter (*α*) represents the diminishing sensitivity to changes in values as the absolute value increases (if *α* < 1). The risk preference parameter (*α* < 1) is equivalent to risk aversion in the reward domain and risk seeking in the punishment domain, as demonstrated by the following examples. Imagine a gamble between a probabilistic reward: 50% of £20; 50% of £0 and a sure reward of £10. The objective expected value of the gamble is £10, similar to the certain option. Hence a risk neutral person would be indifferent between these two options. If *α* = 0.8, the gamble would have a subjective value of 5.49, and the certain option would have a subjective value of 6.31, which results in participants being more likely to choose the certain option (i.e., risk aversion). Now imagine a gamble between a probabilistic punishment 50% of -£20; 50% of £0 and a sure punishment of -£10. The objective expected value of gamble is -£10, similar to the certain punishment option. If α = 0.8, the gamble would have a subjective value of -5.49, and the certain option would have a subjective value of -6.31, which results in participants being more likely to choose the gamble option (i.e., risk seeking). The loss aversion coefficient (*λ*) represents the weighting of losses relative to gains, which was set to 1 as we did not have gambles with both positive and negative outcomes. The second component of a prospect theory model is the probability weighting function. Most prospect theory models assume that probabilities are weighted non-linearly. However, we found that the probability weighting parameter (*γ*) did not significantly improve the model fit (S1 Table, we used a 1-parameter probability weighting function [46]: *w*(*p*) = *exp*(−(−*ln*(*p*)^*γ*^))). Hence, probabilities and utilities were combined linearly in the form: *U*(*p*,*O*) = *p* * *v*(*O*). The third component of a prospect theory model is the choice function. The probability of choosing to gamble is given by the logit or soft-max function:

$$F(p,O_{1},O_{2},O_{c})={\left(1+exp[-\mu (U(p,O_{1},O_{2})-U(O_{c}))]\right)}^{-1}$$ Eq 2

where *O*_1_ and *O*_2_ are the outcomes in the gamble option *[p→O*_1_; *(1-p)→O*_2_*]*, and *O*_*c*_ is the outcome of the certain option. The logit parameter *μ* is the sensitivity of the choice probability to the utility difference. In summary, the prospect theory models included the following free parameters: risk preference parameter (*α)* and stochasticity of decision-making according to the inverse temperature parameter (*μ*).

Parametric approach-avoidance decision model: approach-avoidance models were based on the Prospect Theory models, but with an additional component that allows for value-independent influences to choose or not choose gambles. Specifically, Pavlovian parameter (*δ*) were added to the probability of choosing to gamble (Eq 2) as follows:

$$F(p,O_{1},O_{2},O_{c})={\left(1+exp[-\mu (U(p,O_{1},O_{2})-U(O_{c}))]\right)}^{-1}+\delta$$

$$F(p,O_{1},O_{2},O_{c})=max(0,min(F(p,O_{1},O_{2},O_{c}),1)$$ Eq 3

Positive or negative values of the parameter (*δ*) correspond respectively to an increased or decreased probability of gambling without regard to the value of gamble. Other parts of the models were identical to the Prospect Theory models. In summary, the approach-avoidance model included the following free parameters: risk preference parameter (*α*), stochasticity of decision-making according to the inverse temperature parameter (*μ*) and Pavlovian parameter (*δ*).

### Parameter optimisation

For each model, a set of parameters for that model and a sequence of outcomes observed by an individual participant can be used to calculate the probability of gambling on each trial (Eq 2, Eq 3). The full joint probability of gambling, given a set of parameters for a participant, is provided by the product of the probability for each response actually made. Therefore, the likelihood of a set of parameters given the data is defined by the probability of the data given the parameters. For each participant, we took the sum of the log likelihood of the parameters (Eq 4). For each participant and model, we estimated the parameters which maximized this likelihood by using the search function fmincon in Matlab (minimizing the negative of the log likelihood).

$$L(\Theta |y,p,O_{1},O_{2},O_{c})=\sum_{i=1}^{N}y_{i}\log\left(F(p(i),O_{1}(i),O_{2}(i),O_{c}(i),\Theta )\right)+(1-y_{i})log(1-F(p(i),O_{1}(i),O_{2}(i),O_{c}(i),\Theta ))$$ Eq 4

Where, *i* indexes the trial number; *N* is the number of trials; *y*_*i*_ indicates participant choice on trial *i*, (gamble = 1, skip = 0*)*; *Θ* indicates the parameter vector to be estimated; (*p*,*O*_1_,*O*_2_,*O*_*c*_) represent the gamble options on each trial. Parameters were constrained to the following ranges: *α*: [0,1]; *μ*: (0,10]; *δ*: [−1,1]. The fitting was repeated at 200 random seed locations to avoid local minima.

For each key parameter of prospect theory and approach-avoidance models, we explored the possibility of using separate and joint parameters for reward and punishment domains as well as a weighted or linear probability function. Therefore, we fitted each participant’s choice data with 24 models (S1 Table).

### Model comparison and parameter recovery

We used Akaike’s information criterion (AIC) [30] and Bayesian information criterion (BIC) [31] to compare model fits. Both of these represent a trade-off between the goodness of fit and complexity of the model and thus can guide optimal model selection. AIC and BIC were given by *AIC* = −2*logL* + 2*k* and *BIC* = −2*logL* + *klogN* respectively, where L is the likelihood (Eq 4), k is the number of parameter, N is the number of data points. Lower AIC and BIC values imply a better fit to the data. AIC and BIC were calculated for each participant and each model. AIC and BIC for each model (Fig 4; S1 Table) was its sum over all participants. *Pseudo r*^2^ was calculated with the null model in which α, μ and δ were restricted to 0 ($pseudo\;r^{2}=1-\frac{ln(\hat{L}(model))}{ln(\hat{L}(null\;model))}$, where $\hat{L}$ = Estimated likelihood).

To justify our model selection, we performed model/parameter recovery analysis [47] and model falsification [33]. To reiterate, the PT model had two key parameters (*α*,*μ*), and AA model had three key parameters (*α*,*μ*,*δ*). If the fitted parameters were reliable, we should be able to take simulated data with known parameters, and estimate those parameters. For both models, we chose parameters to represent “typical participants” and generated simulated responses to participants’ observed outcomes. We used the same process as for the original participant responses to estimate parameters for these simulated responses. S3 Fig shows that the fitted parameters for the PT and AA models (based on 50 simulations for each parameter set) are clustered around the parameters used for data generation, suggesting that the parameters were reliable. As shown in S1 Table, for each key parameter, we also explored the possibility of using separate and joint parameters for gain and loss trials as well as a weighted or linear probability function. First, we found that the models with weighted probability did not fit the data better than their linear probability counterpart (based on pseudo r^2^) even with one extra free parameter. Hence, there is no strong evidence in our data set for this free parameter. Second, we compared models with and without separate gain and loss parameters. Fig 4 suggests we have the strongest evidence for using separate δ for gains and loss trials, but weaker evidence for separate α and μ. We also ran a likelihood ratio test for each individual, which compares the goodness of fit of the null model: joint parameter and the alternative model: separate parameters. The test decides whether or not to reject the null model. We found that that using separate δ explained an additional 9391 participants (p<0.05), an extra 2596 participants were explained with separate α, and an additional 1375 participants were explained with separate μ. Finally, Fig 5 justifies our preference for the AA model (α, μ, δ+, δ-) relative to the PT model as it clear that the PT model was unable to generate the behavioural pattern observed (model falsification; [33]). Therefore, all model selection analysis supports our conclusion that the AA model ID = 10 (α, μ, δ+, δ-) is the most likely model.

## Supporting information

S1 Fig Additional participant demographics and screen sizes of the devices used. (a) Age demographic within each gender; (b) Age demographic within each level of education; (c) Percentage of participants in each age group; (d) Percentage of male/female participants; (e) Percentage of participants in each level of education; (f-k) Histograms of the screen size (inches) used within each age group; the screen size were binned as [4, 6, 8, 10]; (i) The control study (Experiment 2) was performed across campus (University of Birmingham) with the first 60 participants (10 per group) using their own mobile device and the second 60 participants (10 per group) using a device provided with a screen size of 5.1inches. Screen sizes of the devices used for first set of 60 participants had a similar profile as in the main experiment (Fig 1).
(TIF)

S2 Fig Movement time and precision during the successful trials. (a-g) Success rate (%) for executing the tapping action given age (as indicated by the legend), screen size (x-axis) and target-size (1: largest target size; 7: smallest target size). Bars/Dots and error bars represent medians and bootstrapped 95%Cis; (h-n) Movement time (seconds) when executing successful tapping actions given age, screen size and target-size; (o-u) Precision (maximal error) of successful tapping actions given age, screen size and target-size. Specifically, during the successful trials, we calculated the radial distance between each of the tapping points and each of the 5 targets; we then took the maximum distance out of these five as our measure of precision. The y-axis represents the ratio of this maximum distance to the radius of the target size, indicating how concentrated these tapping points were around the target. For example, 0.5 meant the tapping position was half way between the centre of the target and the target boundary; whilst 1 meant the tapping position was on the target boundary.
(TIF)

S3 Fig Examples of functions from the decision-making model used to quantify choice behavior. (a) The value function with representative alpha values (as shown in the legend). The x-axis represents objective value, and the y-axis represents subjective value (y = x^alpha). A smaller alpha value indicates reduced sensitivity with increasing value; (b) The weighted probability function with representative gamma values (as shown in the legend). X-axis is the objective probability, and the y-axis represents subjective probability (y = exp(-(-log(probs)).^gamma)).
(TIF)

S4 Fig Model/parameter recovery analysis. (a-d) The approach-avoidance (AA) model had three key parameters: risk preference parameter (*α*); approach-avoidance parameter (*δ*), temperature parameter (*μ*). If the fitted parameters were reliable, we should be able to take simulated data with known parameters, and estimate those parameters. Therefore, we chose parameters to represent “typical participants” and generated simulated responses to participants’ observed outcomes. We used the same process as for the original participant responses to estimate parameters for these simulated responses; (a) The three parameters in 3D space. The black crosses (+) represent the parameter sets used to generate the simulated data, and the *x* symbols represent the best parameter fits found for the simulated data. This shows that the fitted parameters (based on 50 simulations for each parameter set) are clustered around the parameters used for data generation, suggesting that the parameters were reliable; (b) The parameter pair α and μ; (c) The parameter pair α and δ; (d) The parameter pair δ and μ; (e) Parameter recovery for the Prospect theory model. Two key parameters: risk preference parameter (*α*) and temperature parameter (*μ*); (f-h) The histograms of the best-fit parameters for the model with separate parameters for α, μ and δ [*α*+,*α*−,*μ*+,*μ*−,*δ*+,*δ*−]. As shown in (h), there is clear difference between the approach-avoidance parameter (*δ*+,*δ*−) in the gain and loss domains when two separate parameters (as indicated by the legend) were allowed. The difference between gain and loss were weaker for the risk preference parameter (*α*+,*α*−) and the temperature parameter (*μ*+,*μ*−). Quantitatively, we also ran a likelihood ratio test for each individual under the null hypothesis of using a single parameter for gains and losses, and found that using separate δ explained 9391 extra participants (p<0.05), an extra 2596 participants when using separate α, and an extra 1375 participants when using separate μ.
(TIF)

S5 Fig Change in approach-avoidance model parameters across life span for each gender. Column 1 from left: α across age groups; Column 2: δ^−^ and δ^+^ across age groups; Column 3: μ across age groups; Column 4: age-related decline across the punishment and reward domain. The largest effect size was observed for the Pavlovian approach parameter (δ^+^); Bars and error bars represent medians and bootstrapped 95%CIs.
(TIF)

S6 Fig Change in approach-avoidance model parameters across life span for each education level. Column 1 from left: α across age groups; Column 2: δ^−^ and δ^+^ across age groups; Column 3: μ across age groups; Column 4: age-related decline across the punishment and reward domain. The largest effect size was observed for the Pavlovian approach parameter (δ^+^); Bars and error bars represent medians and bootstrapped 95%CIs.
(TIF)

S7 Fig Change in approach-avoidance model parameters across the life span for the Economic decision making task (from Rutledge et al., 2016). (a) Risk preference parameters (α^+^ and α^-^) across age groups; (b) Pavlovian parameters (δ^−^ and δ^+^) across age groups; (c) The temperature parameter (μ) across age groups.
(TIF)

S8 Fig Correlation between motor and economic decision-making tasks for the main approach-avoidance model parameters within each age group. This relationship was relatively consistent across the lifespan whereby we found a positive correlation between these parameters within each age group. However, although the oldest age group (60+) showed a similar trend, we did not have enough power (participant numbers) to reliably detect effect sizes of 0.05 within this group. Specifically, whilst the 60+ age group (n = 783) had 0.28 power to detect 0.05 effect sizes, the next oldest group (50–59, n = 1541) had near double the amount of power of 0.5. Note, the single α parameter of the motor decision-making model was correlated with both the α- and α+ parameters of the decision-making model. Error bars reflect bootstrapped 95% CIs.
(TIF)

S9 Fig Motor decision-making approach-avoidance parameter values median split by economic parameter values within each age group. Filled bars denote participants with below-median values in the economic gambling task; Hollow bars for above-median. The participants with above-median risk parameters and Pavlovian parameters in the economic decision task had generally higher risk parameters and Pavlovian parameters in the motor gambling task. Bars/error bars reflect medians/bootstrapped 95%CIs.
(TIF)

S10 Fig Comparing participants within Experiment 2. This experiment was conducted across campus of the University of Birmingham with the first 60 participants (10 in each age group, the left panel) using their own mobile device and the second 60 participants (10 in each age group, the right panel) using a device provided with a screen size of 5.1 inches. Screen sizes of the devices used for first set of 60 participants had a similar profile as in the main experiment (S1 Fig and Fig 1); (a) The probability estimate performance for the first half participants. For each participant (represented as a dot), the estimation error was calculated as the median error (on each trial: estimate % - 100% if successful, 0% if failed) across all 42 trials. Red crosses and error bars represent the means and 95%CIs across the participants in each age group; (b) The probability estimate performance for the first half participants. Importantly, we did not find a significant difference in estimate performance with or without screen size control. Specifically, an independent-samples t-test was conducted to compare the estimate error in with or without screen size control conditions. There was no significant difference in the estimate error for without (M = -0.023, SD = 0.126) and with screen size control (M = -0.011, SD = 0.139) conditions; t(118) = -0.515, p = 0.6073.
(TIF)

S11 Fig Model results when probability of success was based on individual’s own performance. Similar results are observed when the probability of success was estimated based on each individual’s own data (i.e. the probability of success for a participant facing a certain target size was estimated using their own success rate over the same target size); (a) α across age groups; (b) δ^−^ and δ^+^ across age groups; (c) μ across age groups; (d) age-related decline across the loss and gain domain. The largest effect size was observed for the Pavlovian approach parameter (δ^+^); (e) positive correlation across motor and economic decision tasks for the main approach-avoidance model parameters; (f) median split. Filled bars denote participants with below-median values in the economic gambling task; Hollow bars for above-median. The participants with above-median risk parameters and Pavlovian parameters in the economic decision task had higher risk parameters and Pavlovian parameters in the motor gambling task. Bars/error bars reflect medians/bootstrapped 95%CIs.
(TIF)

S1 Table Comparison of decision-making models. The main parameters were (1) value function parameter (*α*); (2) the probability weighting parameter (*γ*); the Softmax temperature parameter (*μ*); the Pavlovian parameter (*δ*). For each key parameter of prospect theory (PT) and approach-avoidance (AA) models, we explored the possibility of using separate and single parameters for reward and punishment domains as well as a weighted or fixed probability function (see Methods). According to BIC and AIC model comparison an approach-avoidance decision model (ID = 10; bold) fitted the choice (gamble) data better than established decision models based on prospect theory. The preferred model’s behavioural predictions among both the prospect theory models ([*α*^+^,*α*^−^,*μ*^+^,*μ*^−^]; ID = 4) and the approach-avoidance models *([α*,*μ*, *δ*^+^,*δ*^*−*^*];*ID = 10) are plotted in Fig 5.
(TIFF)

S2 Table Statistical power for each parameter in each age group for the correlation between motor and economic decision-making data. Calculated using G-power (http://www.gpower.hhu.de/).
(TIF)
