# Supplementary figures and images for: Age-dependent Pavlovian biases influence motor decision-making
Source: PLoS Comput Biol. 2018 Jul 6;14(7):e1006304. doi: 10.1371/journal.pcbi.1006304 (PMC6051643; doi:10.1371/journal.pcbi.1006304)

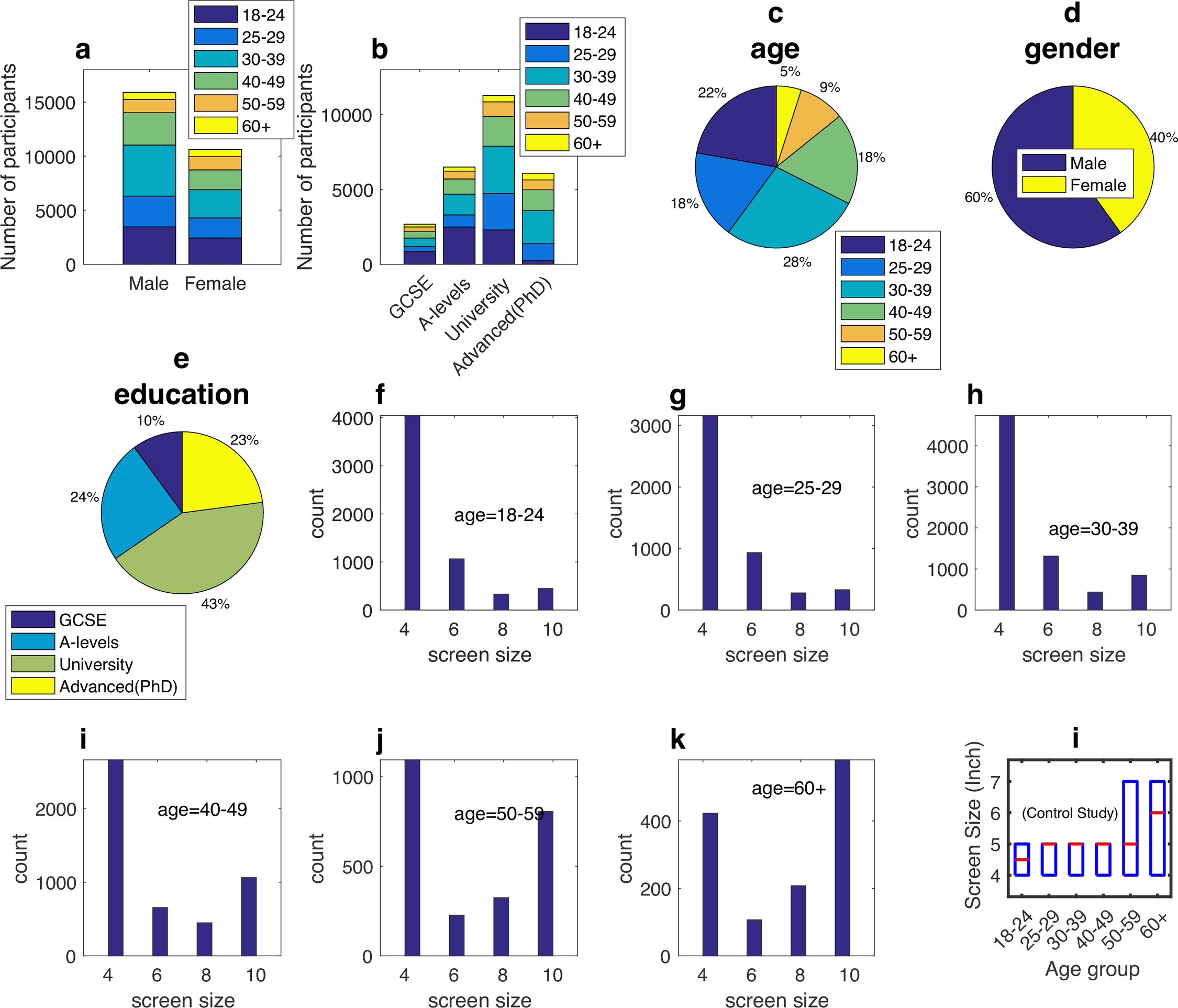

Supplement: S1 Fig — (a) Age demographic within each gender; (b) Age demographic within each level of education; (c) Percentage of participants in each age group; (d) Percentage of male/female participants; (e) Percentage of participants in each level of education; (f-k) Histograms of the screen size (inches) used within each age group; the screen size were binned as [4, 6, 8, 10]; (i) The control study (Experiment 2) was performed across campus (University of Birmingham) with the first 60 participants (10 per group) using their own mobile device and the second 60 participants (10 per group) using a device provided with a screen size of 5.1inches. Screen sizes of the devices used for first set of 60 participants had a similar profile as in the main experiment (Fig 1). (TIF) [file pcbi.1006304.s001.tif]

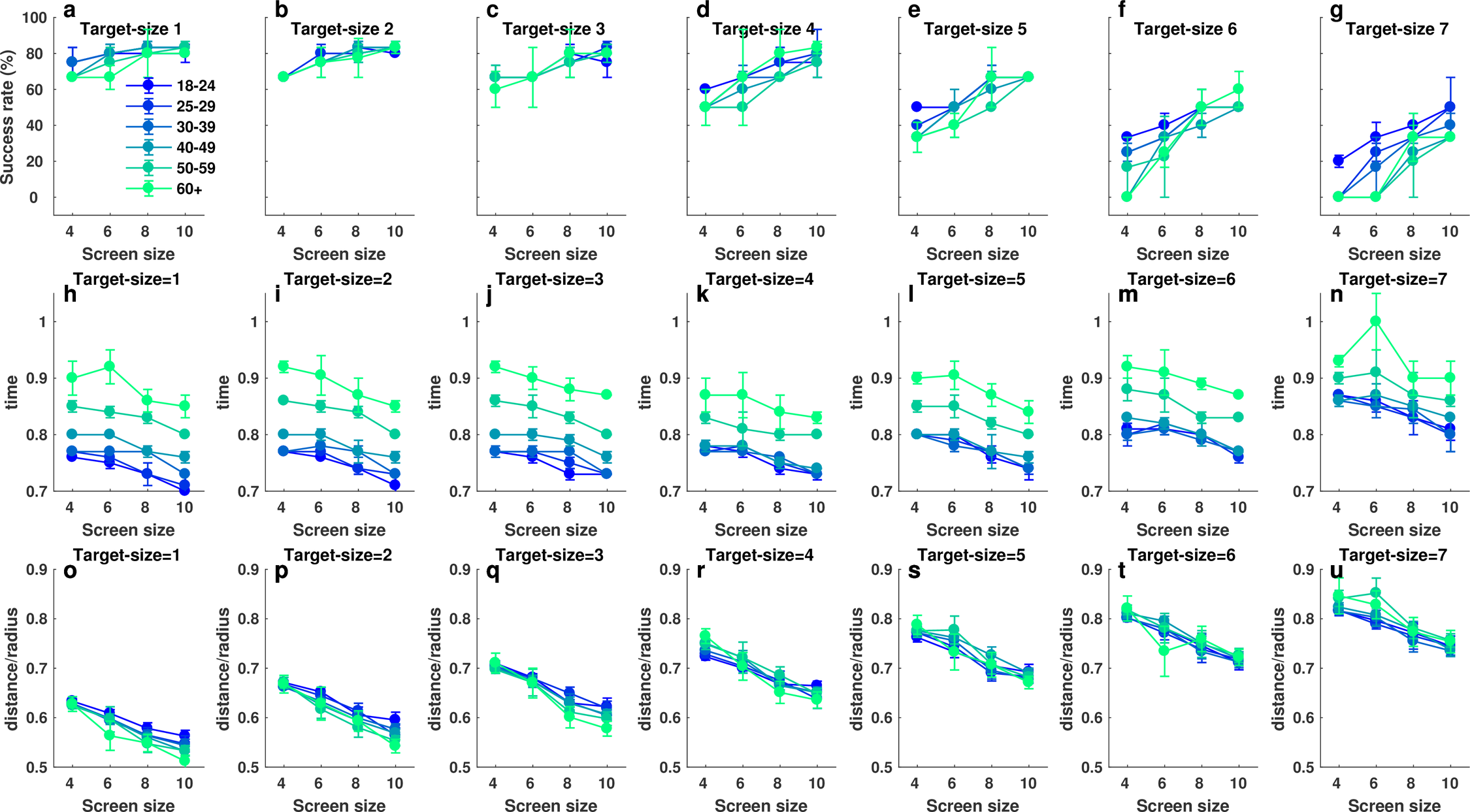

Supplement: S2 Fig — (a-g) Success rate (%) for executing the tapping action given age (as indicated by the legend), screen size (x-axis) and target-size (1: largest target size; 7: smallest target size). Bars/Dots and error bars represent medians and bootstrapped 95%Cis; (h-n) Movement time (seconds) when executing successful tapping actions given age, screen size and target-size; (o-u) Precision (maximal error) of successful tapping actions given age, screen size and target-size. Specifically, during the successful trials, we calculated the radial distance between each of the tapping points and each of the 5 targets; we then took the maximum distance out of these five as our measure of precision. The y-axis represents the ratio of this maximum distance to the radius of the target size, indicating how concentrated these tapping points were around the target. For example, 0.5 meant the tapping position was half way between the centre of the target and the target boundary; whilst 1 meant the tapping position was on the target boundary. (TIF) [file pcbi.1006304.s002.tif]

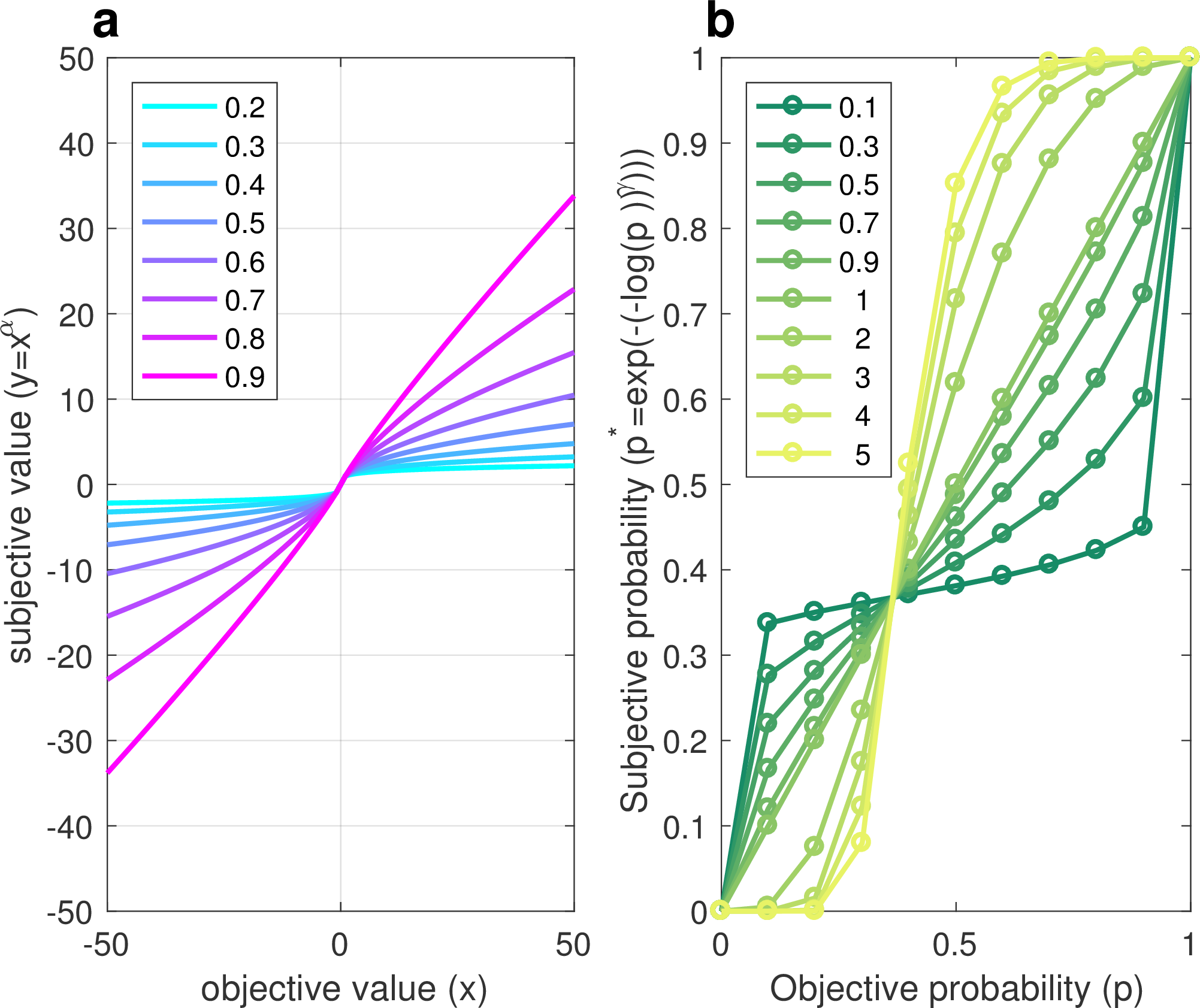

Supplement: S3 Fig — (a) The value function with representative alpha values (as shown in the legend). The x-axis represents objective value, and the y-axis represents subjective value (y = x^alpha). A smaller alpha value indicates reduced sensitivity with increasing value; (b) The weighted probability function with representative gamma values (as shown in the legend). X-axis is the objective probability, and the y-axis represents subjective probability (y = exp(-(-log(probs)).^gamma)). (TIF) [file pcbi.1006304.s003.tif]

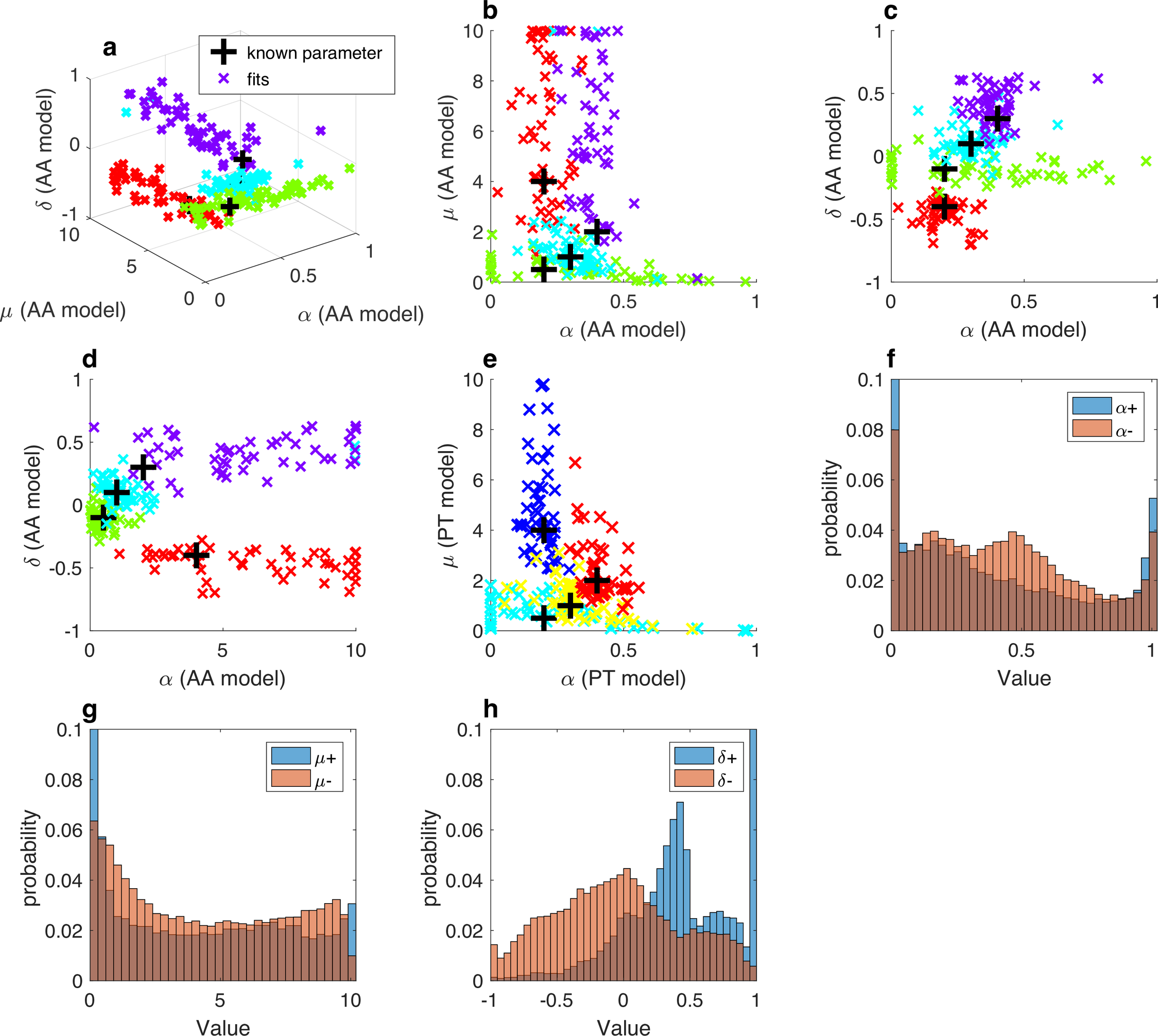

Supplement: S4 Fig — (a-d) The approach-avoidance (AA) model had three key parameters: risk preference parameter (α); approach-avoidance parameter (δ), temperature parameter (μ). If the fitted parameters were reliable, we should be able to take simulated data with known parameters, and estimate those parameters. Therefore, we chose parameters to represent “typical participants” and generated simulated responses to participants’ observed outcomes. We used the same process as for the original participant responses to estimate parameters for these simulated responses; (a) The three parameters in 3D space. The black crosses (+) represent the parameter sets used to generate the simulated data, and the x symbols represent the best parameter fits found for the simulated data. This shows that the fitted parameters (based on 50 simulations for each parameter set) are clustered around the parameters used for data generation, suggesting that the parameters were reliable; (b) The parameter pair α and μ; (c) The parameter pair α and δ; (d) The parameter pair δ and μ; (e) Parameter recovery for the Prospect theory model. Two key parameters: risk preference parameter (α) and temperature parameter (μ); (f-h) The histograms of the best-fit parameters for the model with separate parameters for α, μ and δ [α+,α−,μ+,μ−,δ+,δ−]. As shown in (h), there is clear difference between the approach-avoidance parameter (δ+,δ−) in the gain and loss domains when two separate parameters (as indicated by the legend) were allowed. The difference between gain and loss were weaker for the risk preference parameter (α+,α−) and the temperature parameter (μ+,μ−). Quantitatively, we also ran a likelihood ratio test for each individual under the null hypothesis of using a single parameter for gains and losses, and found that using separate δ explained 9391 extra participants (p<0.05), an extra 2596 participants when using separate α, and an extra 1375 participants when using separate μ. (TIF) [file pcbi.1006304.s004.tif]

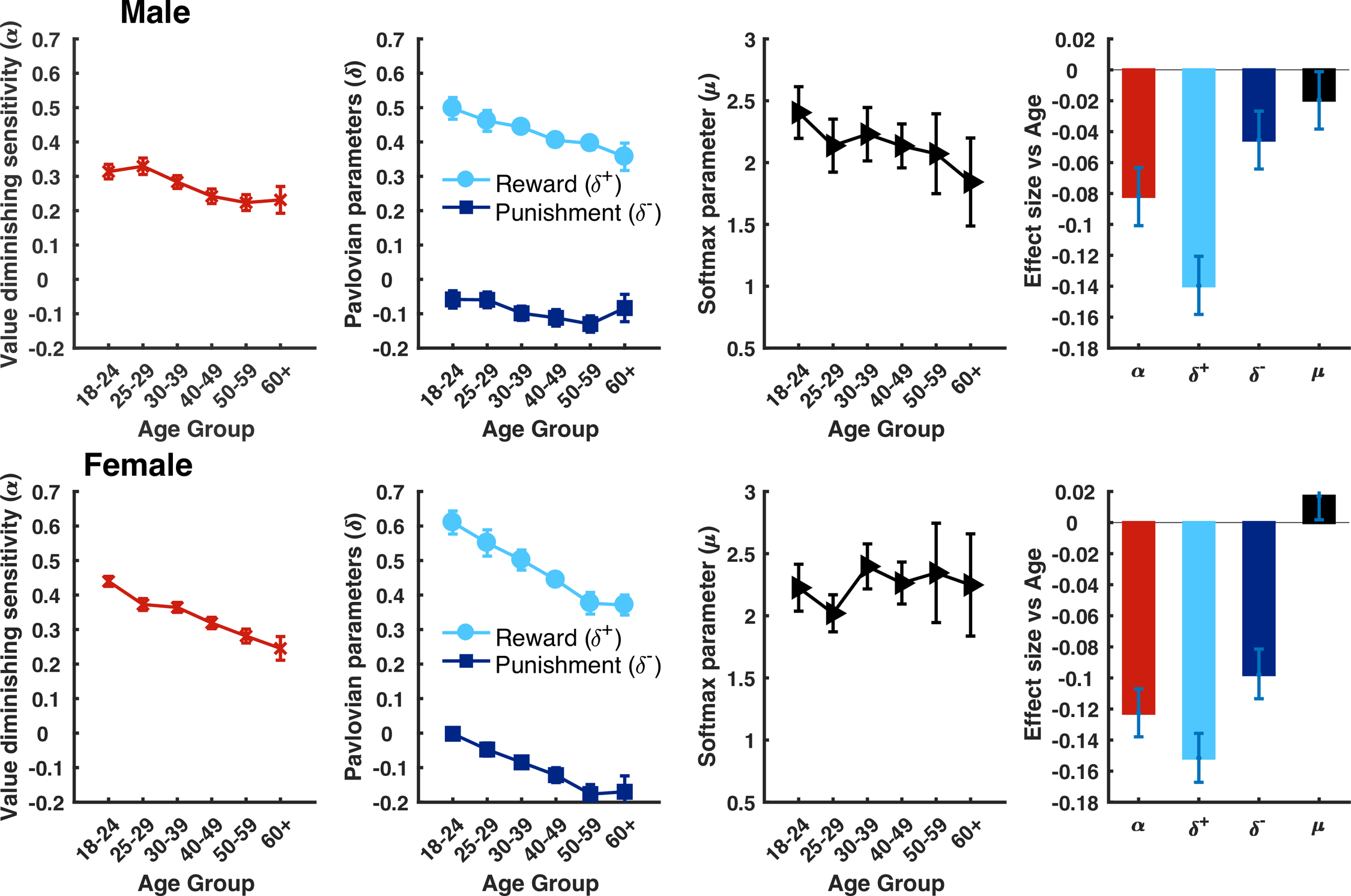

Supplement: S5 Fig — Column 1 from left: α across age groups; Column 2: δ− and δ+ across age groups; Column 3: μ across age groups; Column 4: age-related decline across the punishment and reward domain. The largest effect size was observed for the Pavlovian approach parameter (δ+); Bars and error bars represent medians and bootstrapped 95%CIs. (TIF) [file pcbi.1006304.s005.tif]

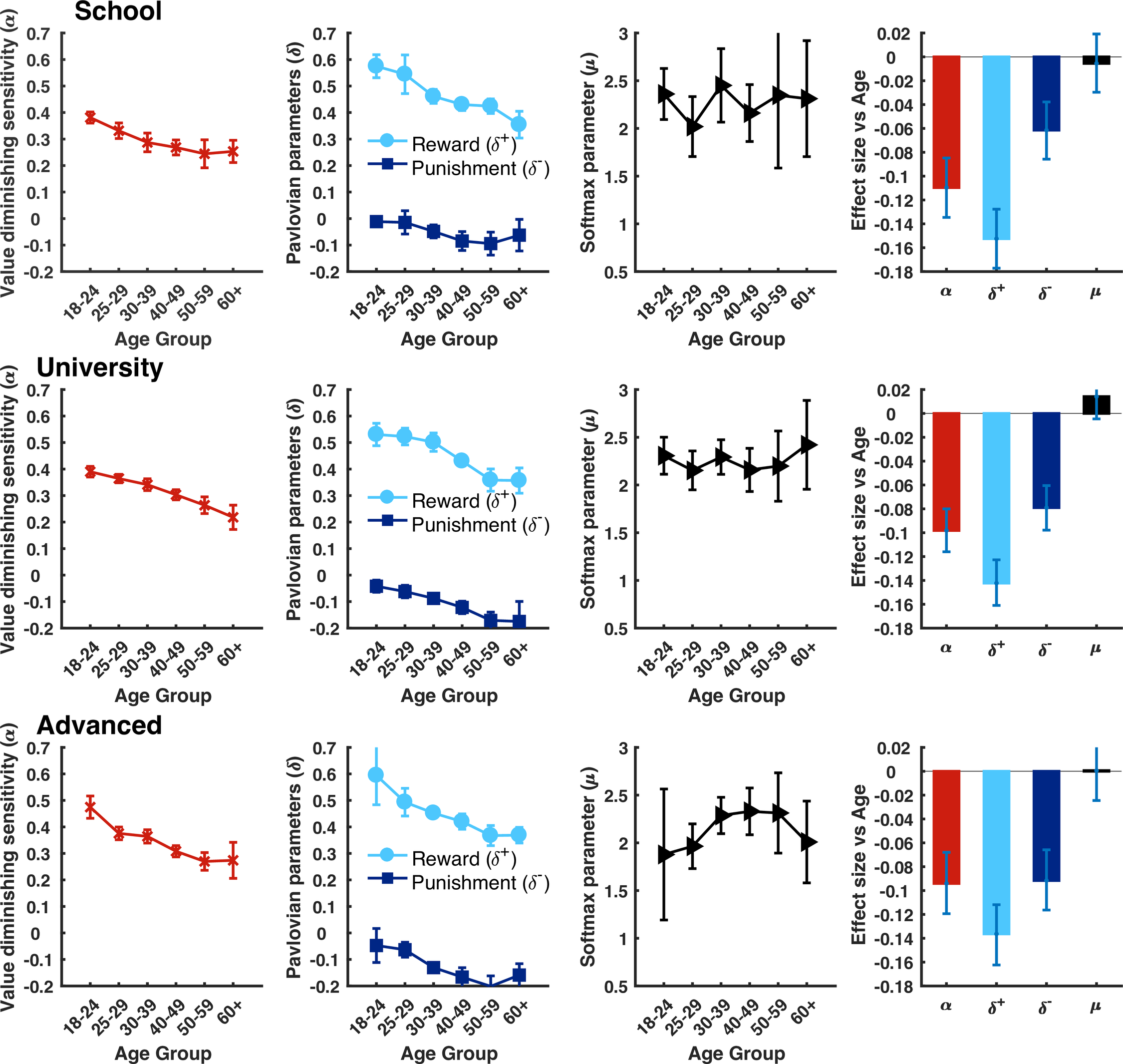

Supplement: S6 Fig — Column 1 from left: α across age groups; Column 2: δ− and δ+ across age groups; Column 3: μ across age groups; Column 4: age-related decline across the punishment and reward domain. The largest effect size was observed for the Pavlovian approach parameter (δ+); Bars and error bars represent medians and bootstrapped 95%CIs. (TIF) [file pcbi.1006304.s006.tif]

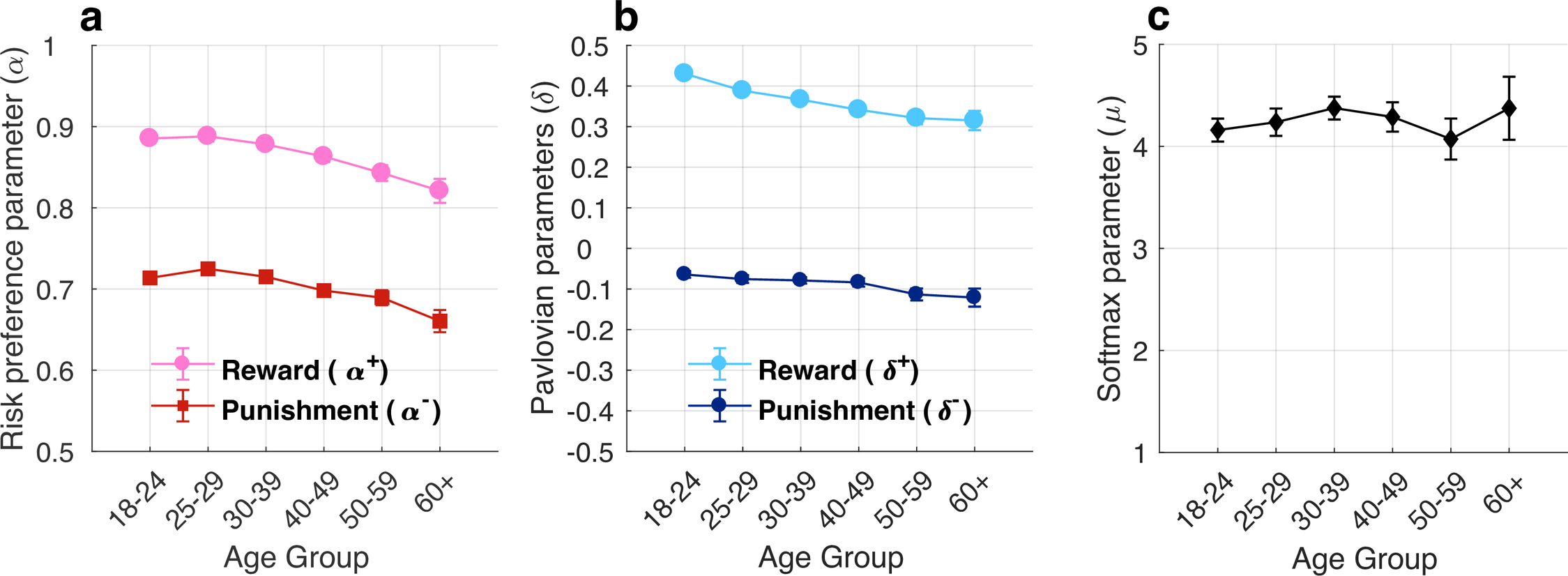

Supplement: S7 Fig — (a) Risk preference parameters (α+ and α-) across age groups; (b) Pavlovian parameters (δ− and δ+) across age groups; (c) The temperature parameter (μ) across age groups. (TIF) [file pcbi.1006304.s007.tif]

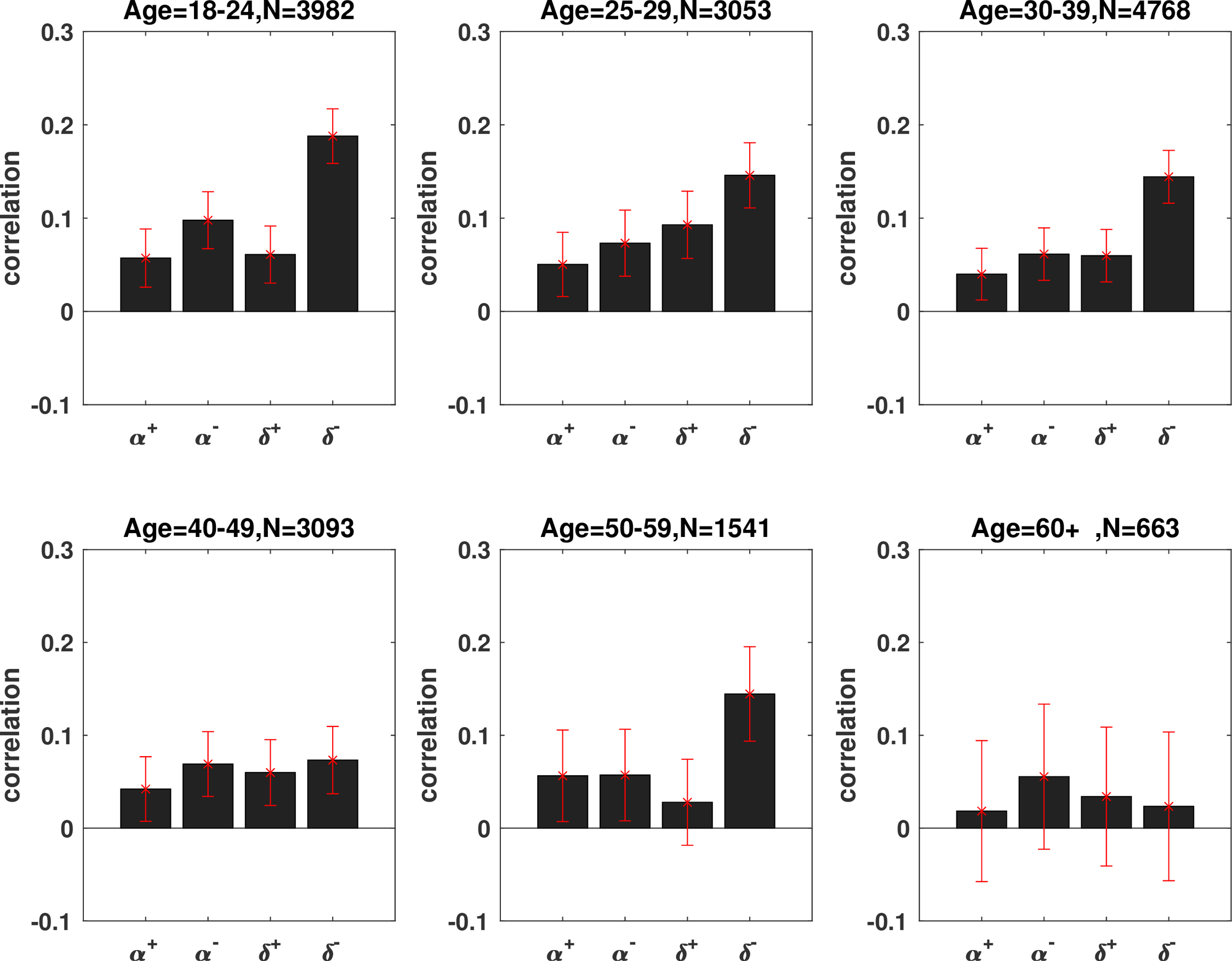

Supplement: S8 Fig — This relationship was relatively consistent across the lifespan whereby we found a positive correlation between these parameters within each age group. However, although the oldest age group (60+) showed a similar trend, we did not have enough power (participant numbers) to reliably detect effect sizes of 0.05 within this group. Specifically, whilst the 60+ age group (n = 783) had 0.28 power to detect 0.05 effect sizes, the next oldest group (50–59, n = 1541) had near double the amount of power of 0.5. Note, the single α parameter of the motor decision-making model was correlated with both the α- and α+ parameters of the decision-making model. Error bars reflect bootstrapped 95% CIs. (TIF) [file pcbi.1006304.s008.tif]

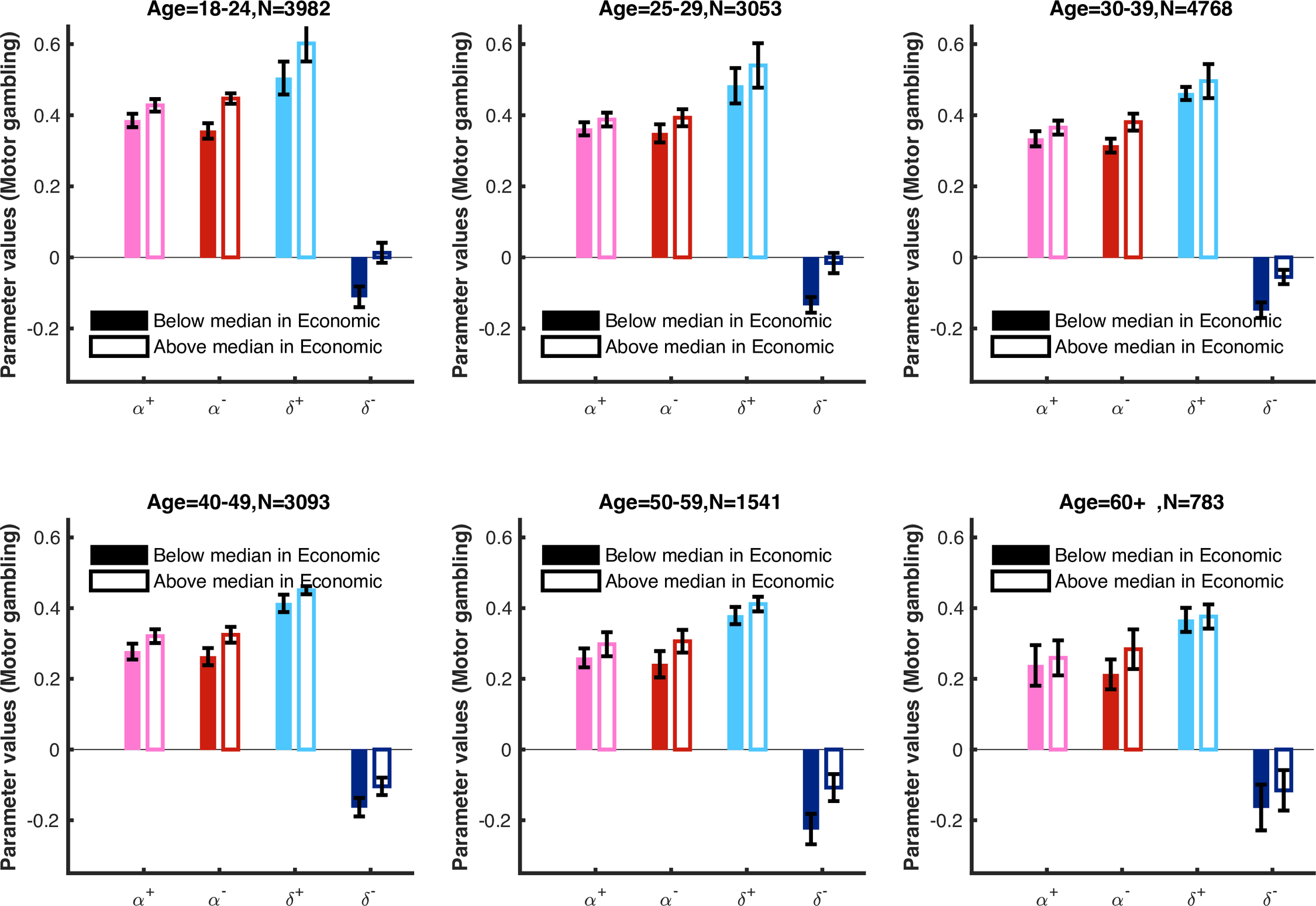

Supplement: S9 Fig — Filled bars denote participants with below-median values in the economic gambling task; Hollow bars for above-median. The participants with above-median risk parameters and Pavlovian parameters in the economic decision task had generally higher risk parameters and Pavlovian parameters in the motor gambling task. Bars/error bars reflect medians/bootstrapped 95%CIs. (TIF) [file pcbi.1006304.s009.tif]

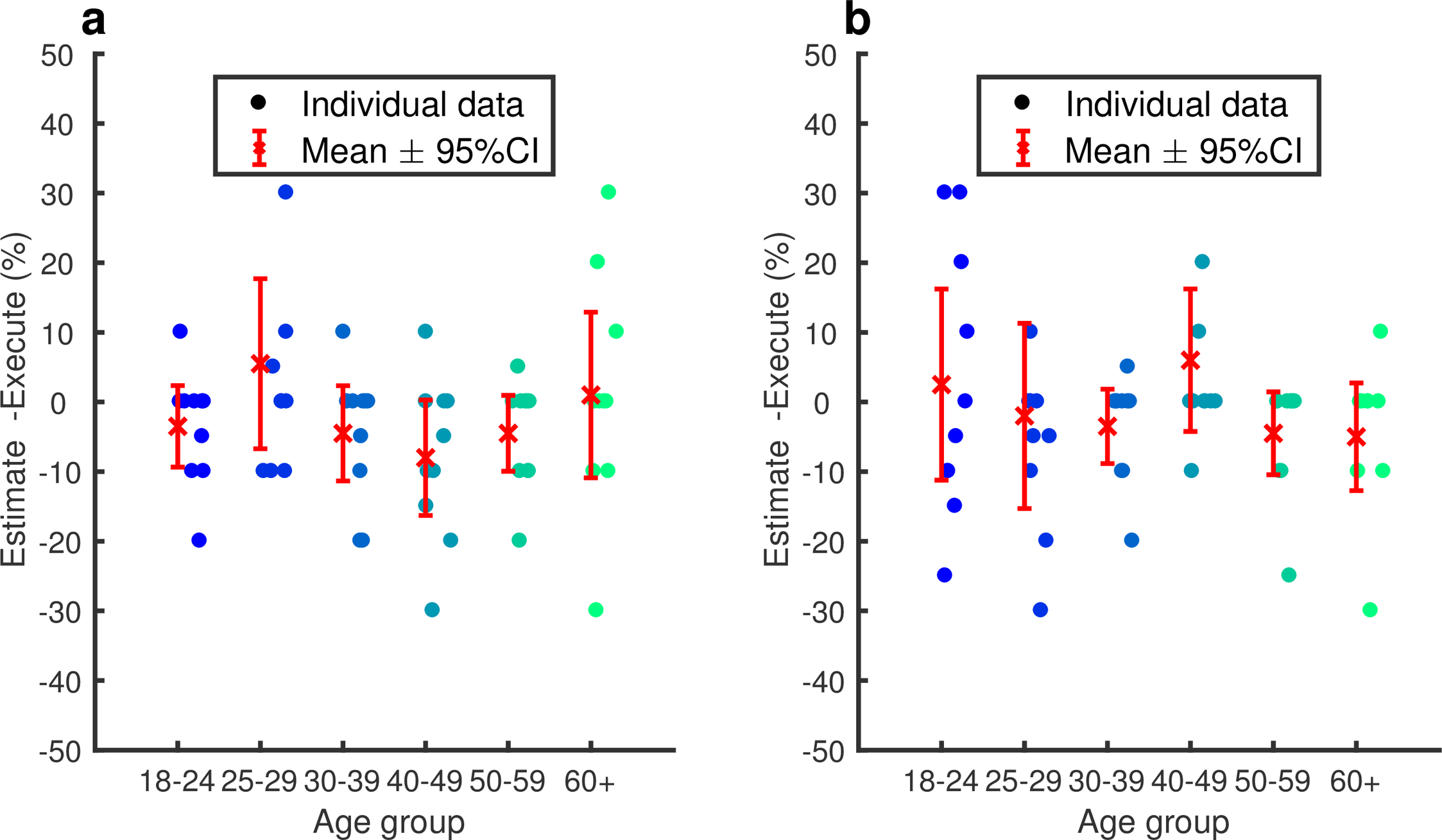

Supplement: S10 Fig — This experiment was conducted across campus of the University of Birmingham with the first 60 participants (10 in each age group, the left panel) using their own mobile device and the second 60 participants (10 in each age group, the right panel) using a device provided with a screen size of 5.1 inches. Screen sizes of the devices used for first set of 60 participants had a similar profile as in the main experiment (S1 Fig and Fig 1); (a) The probability estimate performance for the first half participants. For each participant (represented as a dot), the estimation error was calculated as the median error (on each trial: estimate % - 100% if successful, 0% if failed) across all 42 trials. Red crosses and error bars represent the means and 95%CIs across the participants in each age group; (b) The probability estimate performance for the first half participants. Importantly, we did not find a significant difference in estimate performance with or without screen size control. Specifically, an independent-samples t-test was conducted to compare the estimate error in with or without screen size control conditions. There was no significant difference in the estimate error for without (M = -0.023, SD = 0.126) and with screen size control (M = -0.011, SD = 0.139) conditions; t(118) = -0.515, p = 0.6073. (TIF) [file pcbi.1006304.s010.tif]

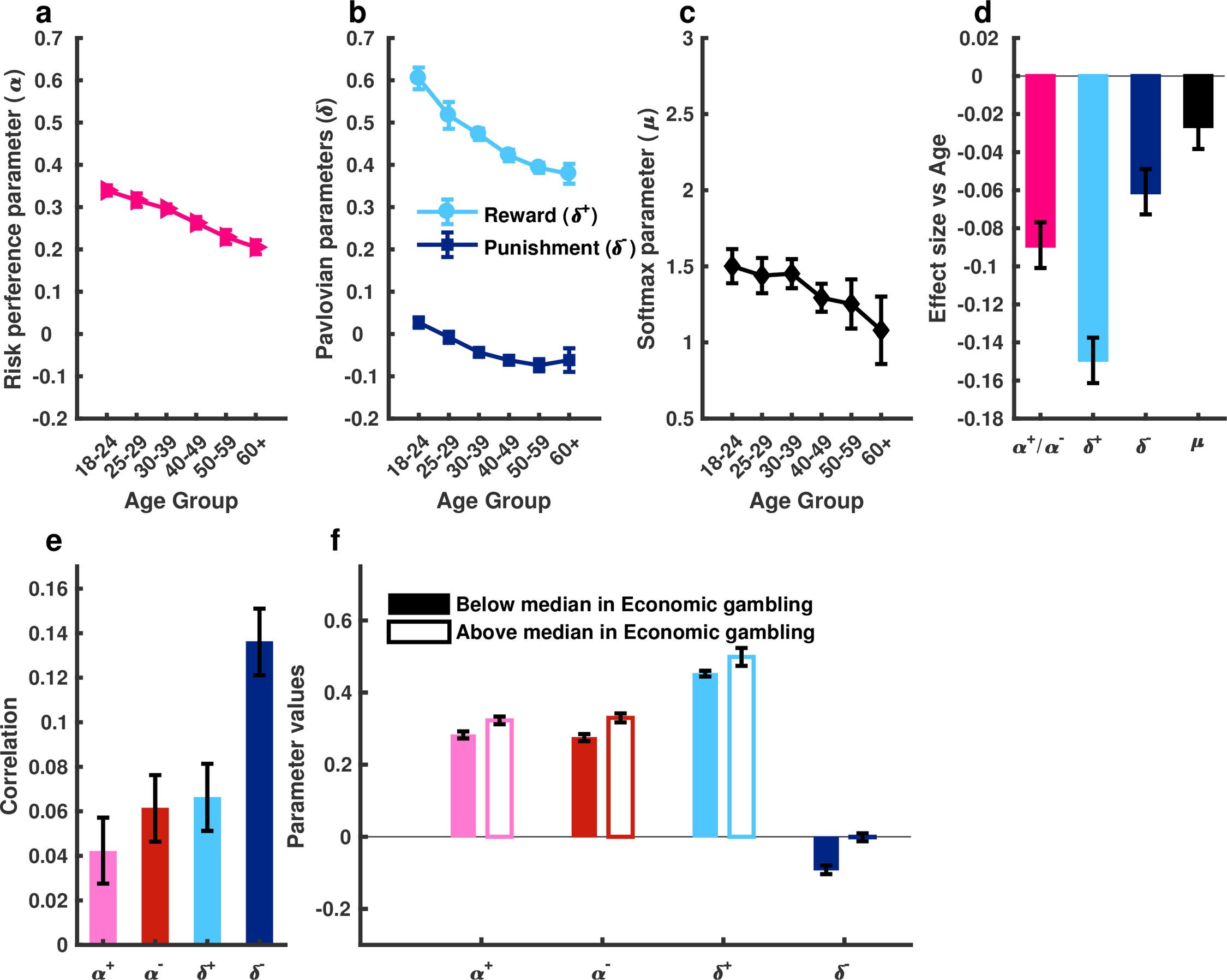

Supplement: S11 Fig — Similar results are observed when the probability of success was estimated based on each individual’s own data (i.e. the probability of success for a participant facing a certain target size was estimated using their own success rate over the same target size); (a) α across age groups; (b) δ− and δ+ across age groups; (c) μ across age groups; (d) age-related decline across the loss and gain domain. The largest effect size was observed for the Pavlovian approach parameter (δ+); (e) positive correlation across motor and economic decision tasks for the main approach-avoidance model parameters; (f) median split. Filled bars denote participants with below-median values in the economic gambling task; Hollow bars for above-median. The participants with above-median risk parameters and Pavlovian parameters in the economic decision task had higher risk parameters and Pavlovian parameters in the motor gambling task. Bars/error bars reflect medians/bootstrapped 95%CIs. (TIF) [file pcbi.1006304.s011.tif]

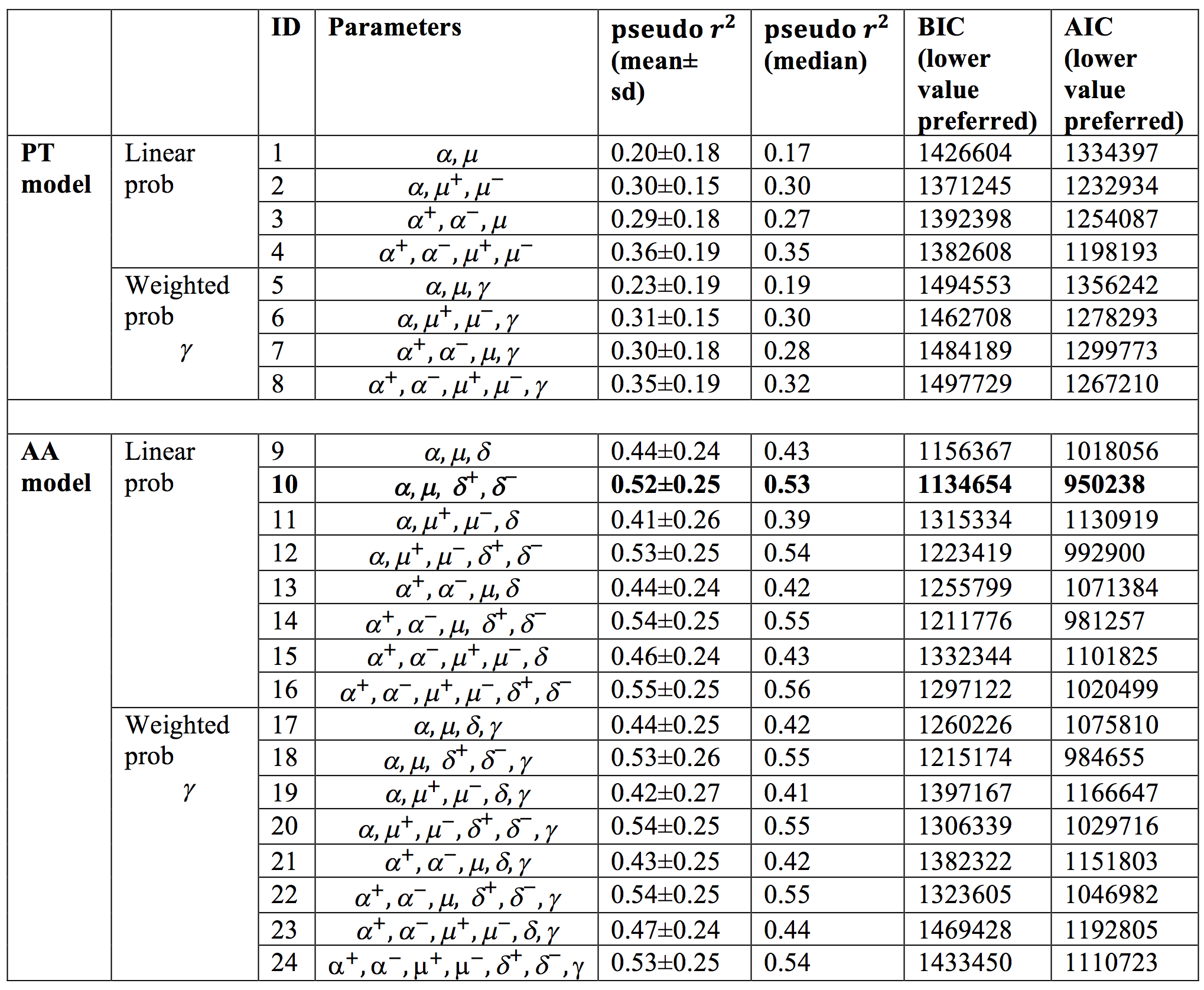

Supplement: S1 Table — The main parameters were (1) value function parameter (α); (2) the probability weighting parameter (γ); the Softmax temperature parameter (μ); the Pavlovian parameter (δ). For each key parameter of prospect theory (PT) and approach-avoidance (AA) models, we explored the possibility of using separate and single parameters for reward and punishment domains as well as a weighted or fixed probability function (see Methods). According to BIC and AIC model comparison an approach-avoidance decision model (ID = 10; bold) fitted the choice (gamble) data better than established decision models based on prospect theory. The preferred model’s behavioural predictions among both the prospect theory models ([α+,α−,μ+,μ−]; ID = 4) and the approach-avoidance models ([α,μ, δ+,δ−];ID = 10) are plotted in Fig 5. (TIFF) [file pcbi.1006304.s012.tiff]

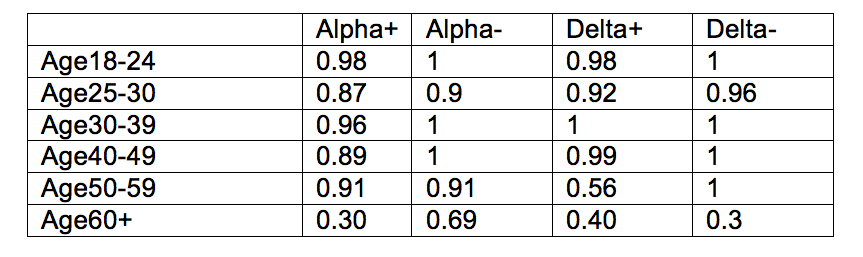

Supplement: S2 Table — Calculated using G-power (http://www.gpower.hhu.de/). (TIF) [file pcbi.1006304.s013.tif]
